# Neoadjuvant Treatment for Penile Cancer: A Systematic Review of Contemporary Evidence

**DOI:** 10.3390/cancers18101595

**Published:** 2026-05-14

**Authors:** Jordan Santucci, Daniel Crisafi, Niranjan Sathianathen, Renu Eapen, Damien Bolton, Declan Murphy, Nathan Lawrentschuk, Marlon Perera

**Affiliations:** 1 Division of Cancer Surgery, Peter MacCallum Cancer Centre, Melbourne, VIC 3052, Australia; 2 Department of Urology, Cabrini Cancer Institute, Melbourne, VIC 3144, Australia; 3 Department of Urology, Queen Elizabeth II Jubilee Hospital, Brisbane, QLD 4108, Australia; 4 School of Translational Medicine, Monash University, Melbourne, VIC 3004, Australia; 5 Department of Urology, Eastern Health, Melbourne, VIC 3128, Australia; 6 Department of Urology, Austin Health, Melbourne, VIC 3084, Australia; 7 Department of Surgery, The University of Melbourne, Melbourne, VIC 3021, Australia; 8 Sir Peter MacCallum Department of Oncology, The University of Melbourne, Melbourne, VIC 3021, Australia; 9 EJ Whitten Prostate Cancer Research Centre, Epworth Healthcare, Melbourne, VIC 3052, Australia; 10 Department of Urology, Royal Melbourne Hospital, Melbourne, VIC 3052, Australia

**Keywords:** penile cancer, penile squamous cell carcinoma, neoadjuvant therapy, chemotherapy, immunotherapy, radiotherapy, targeted therapy, TIP regimen, multimodal treatment, systematic review

## Abstract

**Background/Objectives**: Penile squamous cell carcinoma (SCC) is a rare but aggressive malignancy in which survival declines sharply once regional lymph nodes are involved. Neoadjuvant therapy is recommended for clinically node-positive disease to improve resectability and address micro-metastatic spread; however, the supporting evidence remains limited. We systematically reviewed contemporary data on neoadjuvant strategies for penile SCC, including cytotoxic chemotherapy, radiotherapy, immunotherapy, and molecularly targeted agents. **Methods**: A systematic search of MEDLINE, EMBASE, ClinicalTrials.gov, and CENTRAL was conducted from inception to January 2026 in accordance with MECIR guidance. Eligible studies included patients with histologically confirmed penile cancer treated with neoadjuvant intent prior to curative surgery. Primary outcomes were objective response rate (ORR), pathological complete response (pCR), progression-free survival (PFS), and overall survival (OS). Data were synthesised narratively by treatment modality. **Results**: Forty-two studies met the inclusion criteria (32 chemotherapy, five radiotherapy, five immunotherapy, three targeted therapy). The evidence base was dominated by retrospective cohorts with limited prospective phase II data and no completed randomised trials. Across chemotherapy studies, the median reported ORR was 50% (range 29–90%), with pCR/ypN0 rates ranging 10–25%. Median reported PFS and OS were approximately 11 and 18 months, respectively, with durable survival concentrated among responders undergoing complete surgical consolidation. Radiotherapy data were sparse and heterogeneous. Early-phase immunotherapy combinations reported higher short-term response and pCR signals than historical chemotherapy, though the results were based on small single-arm cohorts. Molecularly targeted systemic monotherapy demonstrated modest activity. **Conclusions**: Neoadjuvant taxane–platinum-based chemotherapy remains the guideline-supported standard for cN2-3 penile SCC, supported by phase II and retrospective data but limited by methodological heterogeneity and absence of randomised evidence. Emerging combination immunotherapy strategies show promising efficacy signals and warrant prospective validation within biomarker-informed trial frameworks.

## Simple Summary

Penile squamous cell carcinoma is a rare but aggressive cancer in which survival outcomes worsen substantially once lymph nodes become involved. For patients with bulky or advanced nodal disease, neoadjuvant treatment given before surgery aims to reduce tumour burden, improve surgical resectability, and treat microscopic metastatic disease early. This systematic review evaluated contemporary evidence for neoadjuvant strategies in penile cancer, including chemotherapy, radiotherapy, immunotherapy, and molecularly targeted therapies. Across 42 included studies, neoadjuvant taxane–platinum chemotherapy regimens, particularly paclitaxel–ifosfamide–cisplatin (TIP), demonstrated objective response rates of approximately 50%, although complete pathological responses were less common. Patients who responded to treatment and subsequently underwent consolidative surgery experienced the most durable survival outcomes. Emerging combination immunotherapy approaches showed encouraging early efficacy signals, while evidence for targeted therapy and radiotherapy remains limited. Overall, the evidence base is constrained by retrospective study designs, heterogeneous methodologies, and the absence of completed randomised trials. Future biomarker-driven prospective studies are needed to optimise treatment selection and improve long-term outcomes for this rare malignancy.

## 1. Introduction

Penile squamous cell carcinoma (SCC) is a rare genitourinary malignancy, with incidence generally reported at <1–2 per 100,000 men in many developed settings, but with marked geographic variation [1,2,3,4]. Despite its rarity, penile SCC carries substantial physical, functional, and psychosocial morbidity, and outcomes deteriorate sharply once the disease progresses beyond the primary tumour [5]. Regional lymph node involvement is a dominant prognostic determinant, with survival falling dramatically in men with bulky inguinal or pelvic nodal metastases, extranodal extension, or distant spread [6]. For advanced presentations, particularly in patients with bilateral inguinal or pelvic nodal disease, poor five-year overall survival (OS) rates of 10–20% have been reported [7]. Clinically node-positive penile SCC presents a critical therapeutic challenge. While surgery remains central to management, pathological findings after lymphadenectomy may reveal high-risk features such as extranodal extension or multiple node involvement that confer a markedly worse prognosis and indicate that surgery alone may be insufficient for durable oncologic control [8]. Moreover, the risk of pathological upstaging after inguinal lymph node dissection highlights limitations in clinical staging and reinforces the need to better identify those who will benefit from early multimodal therapy [9]. Neoadjuvant treatment aims to address both systemic micro-metastatic disease and regional tumour burden to improve resectability before definitive surgical consolidation.

Contemporary international guidelines broadly support a multimodal approach for men with bulky mobile or bilateral inguinal nodal disease (cN2) and fixed inguinal or pelvic lymphadenopathy (cN3), commonly recommending taxane–platinum-based combination chemotherapy for eligible patients administered prior to consolidative lymphadenectomy in responders and those with no evidence of disease progression [6,9,10,11]. Objective response rates (ORRs) with NAC in cN2-3 disease are reported around 50%, with pathological complete responses (pCRs) in the order of ~10–13% [10]. TIP (paclitaxel–ifosfamide–cisplatin) is widely referenced as the standard neoadjuvant regimen, albeit derived from limited prospective phase II evidence and pooled data from predominantly retrospective series [12].

Beyond cytotoxic chemotherapy, novel neoadjuvant strategies in penile SCC are being investigated. Immune checkpoint inhibition has transformed outcomes across several genitourinary malignancies, and there is increasing interest in applying neoadjuvant immunotherapy paradigms to penile SCC based on favourable immune microenvironment features of the disease and efficacy in other squamous cell malignancies [13,14,15]. Concomitant studies have evaluated emerging avenues, including biomarker-driven strategies, molecularly targeted systemic therapies, and antibody–drug conjugates, although clinical data for these approaches in penile SCC remain preliminary [16].

Radiotherapy also remains of interest as a neoadjuvant or downstaging modality in penile cancer, particularly by extrapolation from other squamous cancers, including head and neck, oesophageal, and cervical carcinomas, where chemoradiation is integrated into curative pathways [17,18,19,20]. However, preoperative radiotherapy in penile SCC has not been recommended as a first-line neoadjuvant treatment, given low response rates in early series limited by now outdated techniques [21]. More recent studies of modern chemoradiotherapy report clinically meaningful metabolic and radiological responses in cN3-predominant populations [22].

This review synthesises evidence from a systematic literature search assessing neoadjuvant approaches in penile SCC, including chemotherapy, novel systemic therapies (immunotherapeutics and molecularly targeted agents), and radiotherapy-based strategies. We critically appraise oncological outcomes and toxicity, highlight limitations in the current evidence base, and contextualise findings within contemporary guideline recommendations and ongoing clinical trials.

## 2. Methods

### 2.1. Study Eligibility and Outcomes

The literature was searched systematically in accordance with Cochrane Methodological Expectations of Cochrane Intervention Reviews (MECIR) guidelines [23], with pre-defined inclusion and exclusion criteria (Appendix A). The reporting of the literature search and inclusion of studies was performed in accordance with the PRISMA (Preferred Reporting Items for Systematic Reviews and Meta-Analyses) guidelines, and the literature review has not been registered. Included studies assessed patients with histologically confirmed penile cancer, who received any form of neoadjuvant treatment with the intent for subsequent curative surgical resection of the primary tumour with or without inguinal and/or pelvic lymph node dissection. There were no restrictions on penile cancer histological subtype, stage, grade, or adjuvant treatments after surgery. Studies were excluded if they assessed advanced penile cancer only without curative intent treatment, including patients with metastatic or recurrent disease receiving systemic or radiotherapeutic treatment only. Randomised controlled trials, non-randomised trials, observational studies, case–control studies, and case series were eligible for inclusion in the review. Clinical trial protocols were included to support contextual review even when the trials were still accruing.

The efficacy of neoadjuvant treatment in patients with penile cancer was assessed by evaluating oncological outcomes of any type, with comparison against placebo or control treatments where available. Primary oncological outcomes that were sought included ORR, pCR, overall survival (OS), and progression-free survival (PFS). Secondary outcomes included the safety of neoadjuvant treatments for penile cancer, namely, the incidence of adverse events or dose-limiting toxicity.

### 2.2. Literature Search Strategy, Screening, and Study Selection

A literature search was conducted in Medline and EMBASE from inception to January 2026, with the inclusion of the grey literature, through searches of trial registries on clinicaltrials.gov and the Cochrane Central Register of Controlled Trials, conference abstracts, and unpublished research referenced in clinical practice guidelines or web searches. Hand searches of reference lists from the included studies and relevant guidelines ensured all relevant records were captured. The search was not limited by publication date or language. Two authors (J.S. and D.C.) independently screened abstracts, reviewed full texts of relevant studies, and extracted data. Senior author review was planned to resolve any disagreements.

### 2.3. Strategy for Evidence Synthesis

Data were synthesised through a narrative review according to therapeutic categories. Studies were grouped according to study design and neoadjuvant treatment modality, including chemotherapy, immunotherapy (with or without other treatment), molecularly targeted systemic therapy, and radiotherapy.

## 3. Results

### 3.1. Literature Search and Characteristics of Included Studies

The systematic search identified 471 records from bibliographic databases and trial registries (MEDLINE 108; Embase 61; ClinicalTrials.gov 290; CENTRAL 12). After the removal of 11 duplicate records, 460 studies underwent title and abstract screening. Of these, 386 were excluded for not meeting the inclusion criteria, leaving 74 records for full-text assessment.

Following eligibility assessment, 32 studies were excluded for pre-defined reasons: treatment delivered with primary rather than neoadjuvant intent (n = 5), inability to distinguish neoadjuvant from adjuvant exposure (n = 3), preliminary reports superseded by later included publications (n = 1), radiotherapy delivered with definitive rather than neoadjuvant intent (n = 3), palliative-intent treatment only (n = 5), case reports only (n = 14), or lack of oncological outcome reporting for the neoadjuvant subgroup (n = 1).

**Figure 1:**
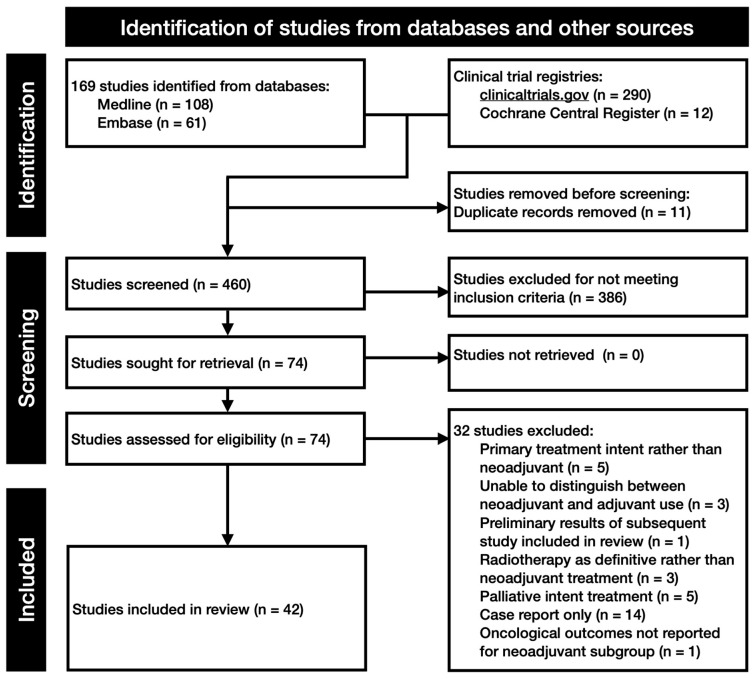
PRISMA flowchart for the selection of studies for inclusion in the review.

In total, 42 distinct studies were included in the final evidence synthesis (Figure 1). These comprised 32 chemotherapy studies, five radiotherapy studies, five immunotherapy studies, and three molecularly targeted therapy studies, noting that several studies evaluated more than one modality and were therefore tabulated across sections. The overall evidence base consisted predominantly of retrospective cohorts and case series, with a smaller number of prospective single-arm phase II trials and ongoing clinical trial that have not yet been completed. There were no completed randomised controlled trials identified.

### 3.2. Evidence Synthesis

#### 3.2.1. Neoadjuvant Chemotherapy for Penile Cancer

##### Study Designs for Neoadjuvant Chemotherapy

Thirty-two studies evaluated NAC for penile SCC (Table 1) [8,24,25,26,27,28,29,30,31,32,33,34,35,36,37,38,39,40,41,42,43,44,45,46,47,48,49,50,51,52,53,54]. Most were retrospective single- or multi-centre cohorts, with two key prospective phase II clinical trials and several comparative retrospective analyses contrasting NAC with adjuvant chemotherapy (AC) or surgery alone. Across studies, NAC was primarily administered to patients with bulky mobile or bilateral inguinal nodal disease (cN2) or fixed inguinal lymphadenopathy (cN3), frequently with pelvic nodal involvement, reflecting a consistently high-risk population. Taxane–platinum-based regimens predominated, particularly taxane–ifosfamide–cisplatin (TIP) and docetaxel–cisplatin–5-FU (TPF) combinations, with older cisplatin-based doublets and triplets used in earlier series. Median numbers of NAC cycles typically ranged from two to four, with radiologic response assessment guiding eligibility for consolidative surgical resection.

**Table 1:** Summary of studies assessing neoadjuvant chemotherapy (NAC) for penile cancer.

Study	Study Design	Study Population Size	Patient and Disease Characteristics	Treatment Details	Median Follow-Up, Months	Outcome Measures	Results
Dhasthakeer et al. (2025) [53]	Retrospective cohort analysis	NAC, n = 38 AC, n = 74	Mean age: 53.5 yrs (range 22–85) SCC (n = 111/112), lymphosarcoma (n = 1/112) Stage: II (n = 11/112), IIIa (n = 34/112), IIIb (n = 62/112), IV (n = 5/112) Baseline characteristics not separately stratified by NAC vs. AC	5-FU + cisplatin: NAC = 28, AC = 60 Paclitaxel+ carboplatin: NAC = 9, AC = 12 Paclitaxel + cisplatin: NAC = 0, AC = 2 Paclitaxel + ifosfamide +cisplatin: NAC = 1, AC = 0 Each given as six cycles, spaced every three weeks	Not reported	ORR (RECIST criteria) DFS CTCAE toxicity	ORR for all NAC regimens = 34/38 (89.5%) 5-FU + cisplatin, PR = 24, PD = 4 Paclitaxel + carboplatin, CR = 6, PR = 3 Paclitaxel + ifosfamide + cisplatin, PR = 1 Median DFS 5-FU + cisplatin, NAC vs. AC = 7 vs. 6 months (p not reported) Paclitaxel + carboplatin, NAC vs. AC = 10 vs. 10 months (p not reported) DFS > 6 months for 5FU + cisplatin vs. paclitaxel+ carboplatin = 40% vs. 85.7% for AC (*p* = 0.024), 52.2% vs. 100% for NAC (*p* = 0.049) Toxicities not significantly different between groups
You et al. (2025) [54]	Retrospective cohort analysis	NAC, n = 11 AC, n = 14	Mean age ± SD: 51.4 ± 10.7 yrs All cTxN + Mx NAC vs. AC: T stage not reported N1 (27.3% vs. 24%), N2 (36.4% vs. 50%), N3 (36.4% vs. 28.6%) (*p* = 0.78) M0 (81.8% vs. 100%), M1 (18.2% vs. 0%) (*p* = 0.78) Comorbidities present: 54.5% vs. 35.7% (*p* = 0.44)	Both NAC and AC received paclitaxel+ ifosfamide + cisplatin q3 weekly LND performed 3–4 weeks after NAC AC started 3 weeks postop	65.1	OS PFS ORR (RECIST criteria)	ORR after NAC = 45.5%; CR = 1/11, PR = 4/11, SD = 5/11, PD = 1/11 NAC vs. AC: Median PFS = 15.7 months (95% CI 10.6–20.9) vs. not reached; NAC inferior HR 3.96 (95% CI 1.04–15.05, *p* = 0.029) Median OS = 75.0 months (95% CI 12.4–137.6) vs. not reached; no significant difference HR 2.01 (95% CI 0.57–7.14, *p* = 0.268) NAC vs. AC status not significant predictor of OS or PFS on multivariate analysis
Noronha et al. (2024) [49]	Retrospective cohort analysis	Curative-intent, n = 71 (NAC, n = 19; AC, n = 36; adjuvant chemoradiotherapy, n = 16) Palliative systemic therapy, n = 20	For curative-intent cohorts: Median age: 57 yrs (IQR 50–65.6) SCC = 70/71, sarcomatoid = 1/71 ECOG PS: 0 (16/71), 1 (48/71), 2 (7/71) cT stage NAC vs. adjuvant: T1 (15.8% vs. 1.9%), T2 (31.6% vs. 50%), T3 (52.6% vs. 48.1%) cN stage NAC vs. adjuvant: N1 (5.3% vs. 13.5%), N2 (63.2% vs. 50%), N3 (26.3% vs. 28.8%), unknown (5.3% vs. 7.7%) Pathological grade NAC vs. adjuvant: Grade 2 (68.4% vs. 65.4%), Grade 3 (31.6% vs. 34.6%)	NAC: all had paclitaxel + platinum (paclitaxel+ cisplatin n = 12/19, paclitaxel + carboplatin n = 7/19) AC: paclitaxel + carboplatin n = 26/36, paclitaxel + cis-platin n = 6/36, 5-FU+ cisplatin n = 1/36, 5-FU+ carboplatin n = 1/36, unknown n = 2 Adjuvant chemoradiotherapy: cisplatin+ radiotherapy n = 15/16, carboplatin+ radiotherapy n = 1/16 Inguinal LND in 60/71 (8/19 in NAC, 52/52 in adjuvant) Pelvic LND in 56/81 (7/19 in NAC, 49/52 in adjuvant)	95	OS ORR (RECIST criteria) R0 resection CTCAE toxicity	ORR after NAC = 37%; CR = 1/19, PR = 6/19, SD = 0/19, PD = 12/19 R0 primary resection after NAC 6/19 (31.5%) Median OS: NAC 14.4 months (95% CI 5.5–23.2) vs. adjuvant treatment 89.9 months (95% CI not reached) vs. palliative 11.4 months (95% CI 9.53–23.3) (*p* < 0.0001) CTCAE toxicities: Overall, well-tolerated. No treatment-related deaths, Grade 3–4 adverse events (mainly haematological) in 25.2%, Grade 3–4 febrile neutropenia in 2.2%
Parrela et al. (2024) [50]	Retrospective cohort analysis	NAC, n = 28 No comparator	Median age: 62 yrs (IQR 44.5–64.5) SCC in all Stage T3/T4 (60.4%) Stage N2/N3 (64.9%)	Paclitaxel, ifosfamide, cisplatin (46.4%) Remaining regimens not specified in meeting abstract	21.8	OS Incidence of disease progression	Median OS = 12.9 months (95% CI 9.8–18.3) Disease progression in 54% Complete response to NAC significantly predicted OS, HR = 0.14, 95% CI 0.02–0.80, *p* = 0.02. CR rate not reported
Soares et al. (2024) [52]	Retrospective cohort analysis	NAC, n = 28 AC, n = 27 Palliative chemotherapy, n = 24	Median age: 62.9 yrs (range 18–103) SCC in all Frequencies of each stage not reported in meeting abstract	Platinum doublet in 95% of cases (not otherwise specified) Remaining regimens not specific in meeting abstract	Not reported	OS PFS ORR (RECIST criteria)	Median OS: NAC 21 months vs. AC 49 months vs. palliative 12 months (*p* < 0.001 adjuvant vs. NAC/palliative) Median PFS: NAC 4 months vs. AC 11 months vs. 1 month palliative (*p* < 0.001 adjuvant vs. NAC/palliative) NAC + surgery versus NAC + no surgery median PFS 6 vs. 2 months (*p* < 0.001) ORR after NAC = 29% (CR = 0%; PR = 29%; SD = 22%; PD = 48%)
Rose et al. (2024) [51]	Retrospective cohort analysis	NAC, n = 209 No comparator	Median age: 60 yrs (IQR 49–67) SCC = 176/209, basaloid = 19/209, sarcomatoid = 3/209, verrucous = 7/209, warty = 4/209 ECOG PS: 0 (117/209), 1 (77/209), 2 (11/209), 3 (4/209) All had locally advanced, clinically node-positive (cTany, cN+), non-metastatic (M0) disease, treated with ≥1 dose NAC before planned lymphadenectomy and primary tumour resection Pathological grade: Grade 1 (13%), Grade 2 (41%), Grade 3 (43%) Stage: II (7%), III (48%), IV (45%)	Paclitaxel, ifosfamide, cisplatin, n = 152 (73%) Carboplatin + taxol, n = 24 (11%) Cisplatin+ taxol, n = 26 (12%) Cisplatin+ 5-FU, n = 7 (3%) Median cycles: four (IQR 3–4) LND in 201/209 (97%)	39	OS PFS ORR (RECIST criteria) Pathologic lymph node complete response (ypN0) CTCAE toxicity	ORR after NAC = 57.2% (PR 87/209 [43.2%], CR 28/209 [13.9%]) Median OS (whole-cohort) = 37.0 months (95% CI 23.8–50.1) Median PFS (whole-cohort) = 26.0 months (95% CI 11.7–40.2) Responder vs. non-responder outcomes: Median OS = 73.0 vs. 17.0 months (*p* < 0.01) Median PFS = 50.0 vs. 11.0 months (*p* < 0.01) Pathological lymph node complete response (ypN0) = 24.8% overall (26% for paclitaxel, ifosfamide, cisplatin) Significant predictors of OS: downstaging after NAC no vs. yes (HR 2.16 95% CI 1.47–3.18); RECIST ORR PD vs. CR (HR 6.88, 95% CI 3.16–14.97); post-chemotherapy pathological N stage ypN3 vs. ypN0 (HR 2.98, 95% CI 1.76–5.04); same factors were significant predictors of PFS Grade ≥ 3 toxicity: 35/209 (17%), treatment discontinuation early due to toxicity 14/209 (6.6%), no treatment-related mortality
de Vries et al. (2023) [48]	Retrospective cohort analysis	Neoadjuvant chemoradiotherapy (±surgical resection of residual disease), n = 23 NAC, n = 37 Therapeutic PLND, n = 24 Prophylactic PLND, n = 142	Median age: 65 yrs (IQR 57–71) SCC in all patients pT stage: pT1 13%, pT2 44%, pT3 33%, pT4 4.4% cN stage: cN0 14%, cN1 38%, cN2 18%, cN3 31% Baseline characteristics not stratified by treatment modality	NAC regimens: - Methotrexate, bleomycin, cisplatin. - Cisplatin, 5-FU, docetaxel. - Cisplatin, 5-FU. Neoadjuvant chemoradiation: 33 daily fractions (1.5–1.8 Gy) + mitomycin day 1 + capecitabine w/rad Adjuvant radiotherapy given for high-risk features in n = 41 (multiple +LNs or ENE)	79	Recurrence rate and RFS OS CSS	NAC vs. neoadjuvant chemoradiotherapy HR for recurrence or progression = 0.71 (95% CI 0.37–1.4, *p* = 0.32) Adjuvant radiotherapy did not significantly impact recurrence HR 1.3 (95% CI 0.86–2.0) Whole-cohort median RFS 11 months (95% CI 7.7–18), 97% recurrences within 2 years Whole-cohort median OS 17 months (95% CI 12–22), 5-year OS 33% Whole-cohort median CSS 18 months (95% CI 13–29) Median CSS after recurrence local 22 months, regional 4.4 months, distant 3.1 months Recurrence pattern among those who recurred in NAC subgroup: regional 6 (32%), distant 13 (68%), local 0
Huelster et al. (2022) [24]	Retrospective cohort analysis	Whole cohort, n = 247 Sample size of NAC subgroup not reported	SCC in all patients Tany, cN+, who received LND either prior to, concurrently, or after primary penile tumour resection Frequency of T and N stages and pathological grades not reported in meeting abstract	NAC treatment details not reported	Not reported	OS RFS	NAC correlated with longer RFS (*p* = 0.006) and OS (*p* = 0.01) Median OS, RFS, and HR not reported for NAC subgroup in meeting abstract
Chahoud et al. (2022) [47]	Retrospective cohort analysis	NAC, n = 156 No comparator	Median age: 59 yrs (range 48–67) SCC in all patients ECOG PS: 0–1 (109/156 [70%]), 2 (47/156 [30%]) cTany, cN+ Clinical stage: cIIA-B 12%, cIIIa 16%, cIIIb 22%, cIV 47%	NAC: paclitaxel, ifosfamide, cisplatin, n = 123/156 (79%) Other platinum-based NAC not otherwise specified, n = 33/156 (21%)	Not reported	OS PFS ORR (RECIST criteria)	Median OS 39 months (95% CI 20.9–57.1) Median PFS 23 months (95% CI 10.4–35.5) ORR after NAC = 56%; CR = 7.6%, PR = 48.4%, SD = 23.3%, PD = 20.7% Post-NAC PD predicted worse OS, HR = 2.1, *p* = 0.02
Bandini et al. (2020) [45]	Retrospective cohort analysis	NAC, n = 79 No NAC, n = 255	Median age: 58 yrs (range 48–67) SCC in all cTany, cN+ who underwent inguinal LND (±pelvic LND) and penile lesion resection NAC vs. no NAC cT stage: cT < 2 (38% vs. 35.7%), cT2 (54.4% vs. 50.2%), cT3–4 (7.6% vs. 14.1%), *p* = 0.3 NAC vs. no NAC cN stage: cN1 (13.9% vs. 42.7%), cN2 (50.6% vs. 43.9%), cN3 (35.4% vs. 13.3%), *p* < 0.001 Laterality of clinical inguinal lymphadenopathy, NAC vs. no NAC: unilateral 46.8% vs. 45.1%, bilateral 50.6% vs. 30.2%, *p* = 0.1 Inguinal and pelvic lymph nodes FDG-PET positive more frequently in NAC vs. no NAC groups (19% vs. 0.4%, *p* < 0.001), but only 4.4% of no NAC patients had PET/CT performed vs. 46.8% of NAC patients (significant risk of misclassification bias)	Two most frequent NAC regimens: cisplatin, 5-fluorouracil, taxane (27.8%), 5-fluorouracil, cisplatin (25.35%), not otherwise specified Pelvic LND in addition to inguinal LND in 52.7% Adjuvant also received: chemotherapy n = 108 (32.3%), radiotherapy n = 35 (10.5%)	70	OM OS	Two-year OS rate similar for NAC vs. no NAC: 58% vs. 70%, *p* = 0.08 Subgroup analysis of “NAC-eligible” patients defined as cN2 with inguinal and pelvic FDG-PET positivity or cN3 disease: 2-year OS significantly rate 23% higher with NAC vs. no NAC (*p* = 0.002). Subgroup analysis of “NAC-ineligible” patients, NAC resulted in 17% lower 2-year OS vs. no NAC (*p* = 0.014). Multivariable Cox regression: NAC independently associated with lower OM rates only in eligible patient cohort, HR 0.28, 95% CI 0.13–0.62, *p* = 0.002.
Gondoputro et al. (2020) [46]	Retrospective cohort analysis	Whole cohort, n = 34 NAC, n = 4/34	Median: age 68.5 yrs SCC 61.8%, basaloid 11.8%, warty-basaloid 5.9%, warty 5.9% cT1 35.3%, cT2 35.3%, cT3-4 29.4% cN1-3 35.3% Despite ~30% presenting with locally advanced disease, only 4/34 (12%) received NAC prior to local surgery or LND	NAC regimens not specified in meeting abstract	12.5	CR rate	CR = 1/4 (25% of patients who received NAC) No other outcomes reported specifically for NAC subgroup
Canter et al. (2019) [8] InPACT; NCT02305654	Randomised controlled trial protocol	A total of 14 patients recruited after first year (aiming 400 over 5 years)	SCC in all Any T, N1-3, M0 ECOG ≤ 2 Low disease burden (one mobile lymph node, no high-risk features) Intermediate disease burden (two ipsilateral mobile lymph nodes, no high-risk features) High disease burden (bilateral, pelvic, or fixed lymph nodes OR ≥ 3 lymph nodes involved radiologically OR presence of high-risk CT features)	Surgery alone vs. NAC + surgery vs. neoadjuvant chemoradiotherapy + surgery If received NAC and pathological high-risk, second randomisation to either adjuvant chemoradiotherapy or prophylactic pelvic lymph node dissection + adjuvant chemoradiotherapy NAC = paclitaxel, ifosfamide, cisplatin (4 21-day cycles) Chemoradiotherapy = concurrent cisplatin once a week, radiotherapy 5 days/wk for 5 weeks	Not yet reported	Primary outcome: OS Secondary outcome: DSS, DFS, freedom from locoregional recurrence and distant metastasis, feasibility, toxicity, surgical complications, QoL	Currently recruiting, outcomes not reported
Necchi et al. (2019) [43]	Retrospective cohort analysis	NAC, n = 86 AC, n = 171 Adjuvant radiotherapy, n = 74 No perioperative therapy, n = 358	Median age: 59 yrs (IQR 49–68) SCC 96.2%, SCC with sarcomatoid variant 3.8% All received regional LND ECOG status (n [%]): 0 (277 [65.3%]), 1 (112 [26.4%]), 2 (24 [5.7%]), >2 (11 [2.6%]) pT < 3N0M0, n = 149 (22.5%) pT3-4N0M0, n = 28 (4.2%) pTanyN1-2M0, n = 211 (31.9%) pTanyN3M0, n = 274 (41.4%) Proximal margin involvement, n = 61 (13.1%); vascular invasion, n = 141 (24.1%); extranodal extension, n = 188 (39.4%) Median number of LNs removed, 20 (IQR 13–28) Median positive LN ratio, 11.1% (QR 0–23.5%)	NAC regimens: Cisplatin, 5-FU, n = 22/86 (25.6%) Paclitaxel, ifosfamide, cisplatin, n = 6/86 (7%) Docetaxel, cisplatin, 5-FU, n = 29/86 (33.7%) Cisplatin-based regimen not specified, n = 29/86 (33.7%) AC regimens: Cisplatin, 5-FU, n = 27/171 (15.8%) Docetaxel, cisplatin, 5-FU, n = 26/171 (15.2%); paclitaxel, cisplatin, gemcitabine, n = 1/171 (0.6%); vincristine, bleomycin, methotrexate, n = 34/171 (19.9%); other regimen not specified, n = 83/171 (48.5%)	50	OS RFS	NAC not associated with improved OS or RFS on Cox multivariate analysis (HR for death 1.61, 95% CI 1.04–2.47, *p* = 0.031; HR for recurrence 2.00, 95% CI 1.31–3.07, *p* = 0.001) AC not associated with improved OS or RFS on Cox multivariate analysis (HR for death 0.84, 95% CI 0.58–1.23, *p* = 0.38; HR for recurrence 0.88, 95% CI 0.59–1.31, *p* = 0.53) Adjuvant radiotherapy not associated with improved OS or RFS on Cox multivariate analysis (HR for death 0.99, 95% CI 0.65–1.51 *p* = 0.98L; HR for recurrence 0.93, 95% CI 0.60–1.44, *p* = 0.75) NAC or AC ineffective in Kaplan–Meyer analyses on OS in subgroups of patients with cN0 and cN1-2 (HR not reported, *p* = 0.41 and *p* = 0.01, respectively, but median OS lower with NAC vs. no NAC in cN1-2) Non-statistically significant OS improvement seen in N3 patients (*p* = 0.15 NAC, *p* = 0.12 adjuvant chemotherapy), median OS not reported but higher with NAC vs. no NAC
Xu et al. (2019) [44]	Retrospective cohort analysis	NAC, n = 19 No comparator	Median age: 56.1 yrs (range 35–69) SCC in all cTany, cN3, M0; all had fixed inguinal LN metastasis, 2/19 also had pelvic LN on preop imaging T stage: T1 21.1%, T2 36.8%, T3 36.8%, T4 5.3% Differentiation: well 21.1%, moderate 36.8%, poor 42.1%	All received NAC ifosfamide, docetaxel, cisplatin Responders (PR/CR), n = 12/19: proceeded to penectomy/partial resection + ILND ± PLND Non-responders (SD/PD), n = 7/19: received palliative radiotherapy 55–70 Gy, 1.8 Gy daily	39.6	OS PFS ORR (RECIST criteria) CTCAE toxicity	Median OS 23 months (95% CI 6.1–39.9) Median PFS 11 months (95% CI 6.7–15.3) ORR after NAC = 63.2%; PR or CR = 12/19; SD = 5/19; PD = 2/19 NAC responders vs. non-responders: Median OS 54 (95% CI 22.0–86.0) vs. 15 (95% CI 9.9–20.1) months, log-rank test *p* < 0.001 Median PFS not reported but significantly longer in responders vs. non-responders on log-rank test, *p* < 0.001 Overall, well tolerated. No toxicity-related deaths. Discontinuation due to Grade 4 toxicity (severe myelosuppression) in 1/19. Grade 3 toxicity in 3/19 (myelosuppression 2/19; nausea/vomiting 1/19)
Chipollini et al. (2018) [42]	Retrospective cohort analysis	NAC, n = 69 Adjuvant therapy (chemo. ± radiation or radiation alone), n = 114 LND alone without perioperative therapy, n = 128 NAC + adjuvant, number not stated	Median age: 59 yrs (IQR 49–68) Histological type not specified pN stage: N1 18.2%, N2 18.5%, N3 63.3% Treatment selection varied by nodal burden: most pN2/N3 received systemic therapy (67%), most pN1 received surgery alone (65%) Baseline characteristics not separately detailed for NAC vs. adjuvant vs. LND-alone groups	Most common chemotherapy regimens were cisplatin-based regimens (65.9%) Specific chemotherapy regimens, cycle numbers, and radiotherapy details not otherwise reported NB: long recruitment period 1964–2017, risk of outdated treatment regimens being included	34	OS PFS CSS	LND alone subgroup: 5-year OS 54.8%, CSS 58.9%, PFS 54.8% Perioperative treatment in NAC, adjuvant, or combined setting not significantly associated with PFS/CSS/OS on multivariable analysis. Multivariable HR vs. LND alone: NAC PFS HR 1.52 (*p* = 1.04); CSS HR 1.59 (*p* = 0.091); OS HR 1.52 (*p* = 0.11) Adjuvant therapy PFS HR 0.61 (*p* = 0.062); CSS HR 0.62 (*p* = 0.095); OS HR 0.72 (*p* = 0.202) NAC + adjuvant PFS HR 1.50 (*p* = 0.293); CSS HR 1.61 (*p* = 0.221); OS HR 1.55 (*p* = 0.249)
Necchi et al. (2017) [40]	Retrospective cohort analysis	Group 1: NAC, n = 94 Group 2: AC, n = 78 Group 3: NAC + AC, n = 21 Unknown chemo. timing, n = 8	Median age: 62 yrs (range 35–87) SCC, n = 144/201; sarcomatoid, n = 8/201; verrucous, n = 19/201; warty, n = 5/201; basaloid, n = 1/201; papillary, n = 16/201 ECOG PS: 0–1 (82.1%), ≥2 (4.5%), unknown (13.4%) Staging: Tany, cN+ (n = 162 [80.6%]; NAC = 77/94; AC = 60/78; NAC + AC = 17/21); T3-4, cN0 (n = 39 [19.4%]; NAC = 17/94; AC = 18/78; NAC + AC = 4/21). No metastatic disease any patient at baseline NAC group had higher proportion of cN3 (47.9%) vs. adjuvant group (24.4%) vs. 0 for NAC + AC (*p* < 0.001) NAC + AC had lower rate of pelvic LN involvement (4.8%) vs. NAC (23.4%) or adjuvant group (17.8%), *p* < 0.001	Docetaxel or paclitaxel, cisplatin, 5-FU, n = 103/201 (51.2%) Cisplatin, 5-FU, n = 48/021 (23.9%) Cisplatin, n = 18/201 (9%) Paclitaxel, ifosfamide, cisplatin, n = 7/201 (3.5%) Methotrexate, bleomycin, cisplatin, n = 6/201 (3%) Other regimens at frequencies ≤ 1.5% Treatment regimens not stratified by treatment group Concomitant radiotherapy, n = 43/201: NAC (12/94 [12.8%]), AC (28/78 [35.9%]), NAC + AC (3/21 [14.3%]) (*p* < 0.001 for difference)	Not reported	OS RFS ORR (RECIST criteria) CTCAE toxicity	Median OS months (95% CI): NAC 17.1 (12.5–21.5) vs. AC 105.3 (19.8-not estimable) vs. NAC + AC 18.5 (11.8–30.2), *p* = 0.45 Median RFS months (95% CI): NAC 7.7 (6.4–9.8) vs. AC 32.8 (9.7–132.7) vs. NAC + AC 11.1 (9.5–24.3), *p* = 0.012 ORR after NAC (Group 1 + Group 3) = 54.8%; CR = 19/115; PR = 44/115; SD = 23/115; PD = 26/115; unknown 3/115 Timing of perioperative chemotherapy (i.e., NAC or AC vs. NAC + AC) was not associated with OS on univariable analysis (NAC vs. NAC + AC HR 0.85, 95% CI 0.31–2.31; AC vs. NAC + AC HR 0.60, 95% CI 0.19–1.89, *p* = 0.45) Type of NAC not associated with RFS or OS (cisplatin vs. no platinum and carboplatin vs. no platinum; taxane vs. no taxane; cisplatin vs. other; taxane + cisplatin + 5-FU vs. cisplatin and 5-FU vs. other regimens) CR after NAC associated with significantly longer median (95% CI) RFS and OS vs. no CR after NAC (not reached vs. 8.4 months [6.7–12.3] and not reached vs. 19.8 [15.6–28.2], respectively, with *p* < 0.001 for both) Grade 3–4 toxicities in Groups 1 and 2: thrombocytopenia 1.3–3.2%, neutropenia 7.7–12.8%, anaemia lower in group 2 vs. group 1 (1.3% vs. 10.6%). Higher incidence Grade 3–4 haematologic (33% vs. ~1–10%) and non-haematologic adverse effects in group 3 (higher burden of chemotherapy)
Matsuda et al. (2017) [39]	Retrospective case series	NAC, n = 4 No comparator	Median age: 46 yrs (range 41–62) SCC in all Locally advanced penile SCC with regional LN metastases, no distant metastases pT stage: pT1 (n = 1), pT2 (n = 2), pT3 (n = 1) cN stage: cN1 (n = 1), cN2 (n = 2), cN3 (n = 1) Pelvic node present in 1/4	Cisplatin + 5-FU, n = 2 (two cycles case 1; three cycles case 2) Paclitaxel + ifosfamide + cisplatin, n = 2 (two cycles case 3; five cycles case 4) LAD performed in all cases post-NAC	79	OS DFS ORR (RECIST criteria) Pathological nodal status CTCAE toxicity	All patients alive with no recurrence or metastasis during follow-up (median DFS ~75 months, median OS ~79 months) ORR only reported for paclitaxel + ifosfamide + cisplatin group: PR in both cases pN0 in all four cases Grade ≥ 3 haematologic toxicity occurred in all four patients (neutropenia Grade 4 in 3/4 and Grade 3 in 1/4; thrombocytopenia Grade 3 in 1/4). No Grade ≥ 3 non-haematologic toxicity.
Zargar-Shoshtari et al. (2016) [38]	Retrospective cohort analysis	Unilateral inguinal and ≥1 pelvic LN metastasis, n = 51 (38/51 had unilateral PLND, 13/51 had bilateral PLND) NAC subgroup, n = 9 AC subgroup, n = 25 Adjuvant radiation, n = 20	Unilateral vs. bilateral PLND; median age yrs (range): 64.5 (35.9–82.8) vs. 61 (43.5–74.5) pT stage: T1 (11 vs. 4), T2 (19 vs. 4), T3 (2 vs. 2), Tx (6 vs. 3) pN stage: N3 in all patients Median (range) number of positive inguinal nodes: three (1–6) vs. two (1–8) Median (range) positive pelvic nodes: two (1–12) vs. two (1–9)	Cisplatin + 5-FU, n = 9/51 Cisplatin + 5-FU + docetaxel, n = 14/51 Methotrexate + bleomycin + vinblastine, n = 6/51 Other rarer regimens in remainder of cases receiving chemotherapy	13.3	OS CSS	Multivariate analysis for OS: NAC vs. no additional periop. therapy: HR 0.10 (95% CI 0.02–0.44, *p* = 0.002) AC vs. no additional periop. therapy: HR 0.16 (95% CI 0.06–0.45, *p* = 0.001) Multivariate analysis for CSS: NAC vs. no additional periop. therapy: HR 0.15 (95% CI 0.03–0.70, *p* = 0.02) AC vs. no additional periop. therapy: HR 0.20 (95% CI 0.06–0.62, *p* = 0.01)
Dickstein et al. (2016) [36]	Retrospective cohort analysis	NAC, n = 61 No comparator	Median age: 60.6 yrs (range 24.5–81.4) T stage: Tany included (frequencies not reported) Clinical N stage: N1 (4.9%), N2 (32.7%), N3 (60.6%) All patients had Zubrod performance status 0–2 and were candidates for chemotherapy + surgical resection as appropriate	Paclitaxel, ifosfamide, cisplatin, n = 54 Other chemotherapy regimen, n = 7 (carboplatin + paclitaxel, 5-FU + cisplatin, methotrexate + bleomycin + cisplatin) Median cycles: four (range 1–10)	67	OS RFS ORR (RECIST) Pathological nodal status	ORR after NAC = 65%; CR/PR = 64%; PD = 23.3%; SD = 10%; 1/61 lost to follow-up, not assessed Consolidative surgery able to be performed in 52/61 (85%) Pathological complete nodal response = 16.4% (all pN0 had paclitaxel, ifosfamide, cisplatin) Overall 5-year status: 20/61 (32.8%) alive without disease; 3/61 (4.9%) alive with disease; 32/61 (52.5%) died from disease Median OS (months) by response group: CR/PR 79.9 (*p* < 0.001 vs. PD), SD 41.2 (*p* = 0.045 vs. PD), PD 10.9 Median RFS (months) by response group: CR/PR 25.5 (*p* < 0.001 vs. PD), SD 31.5 (*p* = 0.023 vs. PD), PD 7.0 Five-year OS by response group: CR/PR = 50.1% vs. SD = 25% vs. PD = 7.7% Five-year RFS by response group: CR/PR = 43.2% vs. SD = 25% vs. PD = 7.1% Pathological stage after NAC predicted OS (pN3 vs. pN0 HR 4.46, 95% CI 1.30–15.25, *p* = 0.017) and RFS (pN3 vs. pN0 HR 4.35, 95% CI 1.47–12.93, *p* = 0.008)
Reddy et al. (2017) [41]	Retrospective cohort analysis	NAC, n = 50 No perioperative therapy, n = 132 AC, n = 14 Adjuvant radiation, n = 9	Median age: 62 yrs (IQR 52–71) SCC in all Pathological T stage: pT0 3.3%, pTIS 2.2%, pT1 37.9%, pT2 42.3%, pT3 10.4%, pT4 1.7% Clinical N stage: cN0 43.4%, cN1 24.7%, cN2 15.4%, cN3 15.4% Pathological Grade: Grade 1, 12.1%; Grade 2, 44.5%; Grade 3, 34.1%; unknown, 9.3% Baseline characteristics not separately stratified by treatment NB: high risk of selection bias given NAC correlated with advanced nodal disease (50% of NAC patients had cN3, Cochran–Armitage trend test, *p* < 0.001)	NAC most commonly paclitaxel, ifosfamide, cisplatin Other NAC regimens rarely used: paclitaxel+ carboplatin; methotrexate, vinblastine, adriamycin, cisplatin (MVAC); cetuximab AC and adjuvant radiation were used in small numbers, and regimens were not specified	50.4	RFS	Median RFS 5.7 months Three-year RFS 70% for whole cohort On univariate analysis, NAC associated with worse RFS, HR 2.90 (95% CI 1.76–4.74, *p* < 0.001), but likely confounding from preponderance of advanced nodal disease in NAC group (receipt of NAC likely surrogate for advanced nodal disease in this cohort) AC not associated with RFS (HR 1.94, 95% CI 0.86–3.81, *p* = 0.11) Independent predictors of worse RFS on multivariable analysis: cN3 (HR 3.53, 95% CI 1.68–7.45, *p* = 0.001), ≥3 positive LN (HR 3.78, 95% CI 2.12–6.65, *p* < 0.001), extranodal extension (HR 3.32, 95% CI 1.93–5.76, *p* < 0.001)
Nicolai et al. (2016) [37]	Retrospective cohort analysis	NAC, n = 28 AC, n = 19	Median age: 63 yrs (IQR 41–78) SCC in all patients Tany, cN2-3, M0 ECOG PS: 0–1 in all No prior radiotherapy or systemic therapy T stage rates: not reported N stage NAC vs. AC: N2 (17.9% vs. 26.3%), N3 (82.1% vs. 73.7%) Bilateral nodal disease: 14/28 (50%) NAC vs. 4/19 (21%) AC (risk of selection bias given bilateral +LN correlated with OS *p* = 0.026)	Paclitaxel, cisplatin, 5-FU, n = 8/47 (5/28 NAC; 3/19 AC) Docetaxel, cisplatin, 5-FU, n = 39/47 (23/28 NAC; 16/19 AC) Median (IQR) cycles: three (3–3) NAC vs. four (2–4) AC	22	OS PFS ORR (RECIST criteria) CTCAE toxicity	AC vs. NAC log-rank test for OS *p* = 0.15 and PFS 0.025, with separation of Kaplan–Meier curves in favour of AC (median survival times not reported). Two-year disease-free survival (95% CI) AC vs. NAC: 36.8% (15.2–58.5%) vs. 7.1% (0–16.7%) ORR after NAC = 42.9%; CR = 3.6%; PR = 39.3%; SD = 35.7%; PD = 21.4% Response to NAC (CR + PR vs. SD + PD) was not predictive of OS (*p* = 0.08) or DFS (*p* = 0.07) No dose reductions or delays for toxic events. Neutropenia most frequent Grade ≥ 3 event (25.5%); anaemia 6%, thrombocytopenia 4%, febrile neutropenia 2%. One early (<30 days) death due to cardiac treatment-related toxicity in 1/47 (2.2%). Non-haematologic Grade ≥ 3 events: cardiac 2%, nephrotoxicity 1%, neuropathy 1%, diarrhoea 3%, mucositis 2%, alopecia 13%
Djajadiningrat et al. (2015) [35]	Prospective non-randomised cohort study	NAC, n = 26 No comparator	Median: age 61 yrs (range 35–73) SCC in 92.3%, SCC with sarcomatoid variant in 7.7% Primary inoperable 14/26; recurrent inoperable 12/26 WHO performance status: 0 (10/26), 1 (15/26), 2 (1/26) cT stage: recurrent 7/26 (26.9%), T2 11/26 (42.3%), T4 8/26 (30.8%) cN stage: N0 8/26 (30.8%), N2 3/26 (11.5%), N3 15/26 (57.7%) Differentiation grade: well 3/26 (11.5%), intermediate 11/26 (42.3%), poor 12/26 (46.2%)	Docetaxel, cisplatin, 5-FU, n = 26 All four cycles completed 12/26 (48%), modified cycles 4/26 (15%), stopped early 10/26 (38%; progression 7/26, toxicity 2/26, both 1/26)	30	OS DSS PFS ORR (RECIST criteria) CTCAE toxicity	Median OS (95% CI) 10.1 months (6.7–28.1), 2-year OS (95% CI) 26.9% (14–53%), with significant difference in OS according to NAC response (*p* = 0.0035) Median PFS (95% CI) 7.0 months (1.9–17.1), 2-year PFS (95% CI) 12% (4–33%) Median DSS (95% CI) 10.3 months (8.0—not reached), 2-year DSS (95% CI) 28.4% (15–56%), with significant difference in DSS according to NAC response (*p* = 0.0015) ORR after NAC = 42.3%; CR = 7.7%; PR = 34.6%; SD = 15.4%; PD = 38.5% Curative-intent surgery able to be performed in 14/15 patients with SD or better Considerable toxicity, all patients had some grade of toxicity; Grade ≥ 3 anaemia 2/26, febrile neutropenia 1/26, neutropenia 1/26, abdominal infection 1/26, heart failure 1/26, nausea 3/26, hypokalaemia 1/26, acute kidney injury 2/26, syncope 3/26; 23% discontinued treatment due to toxicity; no treatment-related deaths
Giannatempo et al. (2014) [33]	Retrospective cohort study	NAC, n = 28 AC, n = 19	SCC in all Tany, cN2-N3, M0 (frequencies of T and N stages not reported) Baseline characteristics otherwise not reported (abstract only)	Paclitaxel or docetaxel, cisplatin, 5-FU, n = 47	22	OS PFS ORR (RECIST criteria) Safety	Durable remission more frequent in AC (52.6%, median follow-up 42 months) vs. NAC (28.6%, median follow-up 17 months) No significant differences in OS and PFS on Kaplan–Meier curves between AC and NAC On multivariable analysis, AC was associated with improved OS (*p* = 0.008) and PFS (*p* = 0.002) ORR after NAC = 43% (CR + PR), pathological CR in 4/28 (14%); curative-intent surgery possible in 18/28 (64.3%) Neither OS nor PFS were associated with NAC response Tolerability was mild to moderate
Zou et al. (2014) [34]	Retrospective cohort study	NAC, n = 24 No comparator	Median age: 53.4 yrs (range 38–71) SCC in all T stage: T1 8/24 (33.3%), T2 11/24 (45.8%), T3 5/24 (20.8%) pN3 stage in 24/24 (100%), Tumour differentiation: well 20.8%, moderate 41.7%, poor 37.5%	Bleomycin, methotrexate, cisplatin, n = 24 (mean two cycles, range 1–4) After two cycles, response assessed; non-responders given palliative local radiotherapy 50–70 Gy, responders proceeded with surgery (penectomy/partial amputation + bilateral inguinal LND) + adjuvant BMP 2–4 cycles	50.1 (mean)	OS ORR (RECIST criteria) CTCAE toxicity	Five-year OS: whole cohort 11/24 (45.8%); responders 11/15 (73.3%) vs. non-responders 0/9 (0%), log-rank *p* < 0.001 Significant separation of OS Kaplan–Meier curves in favour of NAC response vs. non-response (median OS not reported) ORR after NAC = 62.5%; CR = 0%; PR = 62.5%; SD = 29.2; PD = 8.3% No treatment-related deaths; main chemotherapy toxicity bone marrow suppression in 83.3% (Grade 3 in 1/24 [4.2%]); oral mucosal damage in 45.8% (11/24), all Grades 1–2. No patients experienced pulmonary fibrosis.
Chiang et al. (2014) [32]	Retrospective case series	NAC, n = 12 No comparator	Median age: 65.5 yrs (IQR 54.8–71.5) SCC in all T stage: T1 4/12 (33.3%), T2 4/12 (33.3%), T3 4/12 (33.3%) N stage: N0 7/12 (58.3%), N1 2/12 (16.7%), N2 3/12 (25%) All M0 Tumour differentiation: good 6/12 (50%), moderate 6/12 (50%)	Intra-arterial chemotherapy via pigtail catheter at abdominal aortic bifurcation, with methotrexate + mitomycin + bleomycin + cisplatin or carboplatin + 5-FU, infused continuously by pump for 2 days in each course, with courses repeated at 4-week intervals Median (IQR) cycles: two (2–3) Residual tumour underwent partial or total penectomy; incisional biopsy to confirm no residual if CR; inguinal LND if palpable LNs or if T1G2 or higher with non-palpable LNs	21	ORR (RECIST criteria) RFS CTCAE toxicity	ORR = 83.3%; CR = 4/12 (33.3%); PR = 6/12 (50%); SD = 2/12 (16.7%); PD = 0/12 (0%); in N0 patients, ORR = 100% (CR = 4/7; PR = 3/7) 4/12 (33.3%) experienced recurrence after median follow-up 27.5 months (range 12–49), 2/4 of whom died of disease (21 and 34 months), RFS significantly longer in LN-negative group (log-rank *p* = 0.041) No discontinuation of IA NAC due to toxicity; Grade 1–2 anorexia was most frequency toxicity (7/12 [58%]); Grade 3 haematological toxicity in 3/12 (25%); no other Grade 3–4 toxicities; no toxicity-related death.
Zhu et al. (2013) [25] Unique Trial Number in UMIN-CTR registry: UMIN 000002697	Phase II prospective, single-arm trial	NAC, n = 23 (locally advanced disease) No comparator Palliative chemotherapy for n = 16 metastatic patients	Meeting abstract only available for data on NAC for locally advanced—minimal baseline characteristic data SCC in all patients Chemotherapy-naïve population No further TNM staging details	Docetaxel, cisplatin, 5-FU, n = 39 Cycle given every 3 weeks Response assessed every two cycles using RECIST criteria	Not reported	Primary: ORR Secondary: safety, PFS, pain response	ORR overall after chemotherapy = 16/39 (41%, 95% CI 27–56.7%); CR = 3/39 (7.7%); PR = 13/39 (33.3%), SD and PD not reported ORR by subgroup: locally advanced 47.8% vs. metastatic 31.3% (*p* = 0.34) Locally advanced consolidation surgery rate = 16/23 (69.6%) Pathologic CR in 2/23 post-surgery Median PFS for NAC locally advanced cohort 15 months (95% CI 6–36) Toxicity (Grades 3–4): neutropenia 46.2%, leukopenia 38.5%, anaemia 12.8%, infection 10.3%; no treatment-related deaths Pain response: 25/39 (64.1%)
Nicholson et al. (2013) [31] Registration on controlled-trials.com: CRUK/09/00, ISRCTN 78108737	Phase II prospective, singe-arm trial	NAC, n = 21 (locally advanced disease [TanyN2-3M0 or T3N1M0]) No comparator Palliative chemotherapy for n = 8 metastatic patients (M1)	Median age: 60.7 yrs (IQR 49.7–65.5) SCC in all patients T stage: TX 11/29 (37.9%), T1 5/29 (17.2%), T2 7/29 (24.1%), T3 6/29 (20.7%) N stage: N0 1/29 (3.4%), N1 5/29 (17.2%), N2 7/29 (24.1%), N3 15/29 (51.7%), NX 1/29 (3.4%) Locally advanced M0 21/29, metastatic M1 8/29 Operability at entry: inoperable 20/29 (69%), operable 9/29 (31%) All were ECOG PS 0–2, and eGFR ≥ 60 mL/min	Three cycles of docetaxel, cisplatin, 5-FU (q21 days) Overall, 21/29 received all three cycles; 5/29 discontinued after two cycles; 3/29 discontinued after one cycle	14.5	Primary: ORR (RECIST criteria) Secondary: operability after chemo., toxicity, PFS, OS	ORR after chemotherapy (evaluable n = 26) = 10/26 (38.5%, 95% CI 20.2–59.4); CR = 2/19 evaluable (10.5%) locally advanced ORR by subgroup: locally advanced = 7/19 (36.8%, 95% CI 16.3–61.6%) vs. metastatic = 3/7 (42.9%, 95% CI 9.9–81.6%) Conversion to operability among inoperable at entry (n = 20) = 5/20 (25%), one too frail for surgery Median PFS 7.1 months (95% CI 2.7–upper limit not reached) Median OS 13.9 months (95% CI 6.1–upper limited not reached) One or more Grade 3–4 toxicities in 19/28 (65.5%); neutropenia 46.4%; febrile neutropenia/sepsis in 6/28 (21.4%); 3/19 (15.8%) had Grade 3–4 toxicities at 3 months (two peripheral oedema; one hypertension); no toxicity-related deaths. Study did not meet prespecified threshold for further research (ORR target 14/29 = 48.3%) and caused significant toxicity; conclusion did not support routine use of docetaxel, cisplatin, 5-FU (but CR and conversion to operability warrants consideration in neoadjuvant setting)
Pagliaro et al. (2010) [30]	Phase II prospective, single-arm trial	NAC, n = 30 No comparator	Median age: 57.5 yrs (range 24–78) ECOG PS: 0 (26.7%), 1 (50%), 2 (23.3%) SCC keratinizing 83.3%, basaloid 13.3%, mixed squamous/verrucous 3.3% Tany, cN2-3, M0 Clinical node stations: groin only (stage III) = 30%, deep/inguinal (stage IV) = 70%	Four cycles of paclitaxel, ifosfamide, cisplatin, q21-28 days Response assessed by CT after cycle 2 and post-treatment using RECIST criteria Consolidation surgery: inguinal ± pelvic LND with resection of residual primary cancer Overall, 76.7% completed four cycles; 7/30 discontinued chemotherapy after 1–3 cycles (rapid PD n = 3; paclitaxel hyper n = 1; cardiac event n = 1; patient’s decision for no further treatment n = 2) No patients received adjuvant radiotherapy	34	ORR (RECIST criteria) Pathologic CR OS PFS CTCAE toxicity	ORR after NAC = 15/30 (50%, 95% CI 31–69%); CR = 3/30 (10%); PR = 12/30 (40%); SD = 9/30 (30%); PD = 6/30 (20%) Consolidation surgery rate = 22/30 (73.3%) Pathologic CR in 3/30 (10% of total; 13.6% of surgical patients who completed TIP regimen) Median PFS 8.1 months (95% CI 5.4–50+) Median OS 17.1 months (95% CI 10.3–60+) Response to NAC associated with significantly longer PFS (log-rank *p* = 0.001) and OS (log-rank *p* < 0.001) PFS and OS significantly worse in those with bilateral residual tumour vs. without (median PFS 5 vs. >50 months, *p* = 0.002, and median OS 10 vs. 36 months, *p* = 0.017, respectively) Well-tolerated; no deaths due to chemotherapy-related toxicity; 16 Grade 3–4 adverse events due to chemotherapy, including infection most frequently (n = 5), with each of the remainder occurring once (neutropenia, anaemia, febrile neutropenia, thrombocyte-penia, myocardial infarction, allergic reaction, CVC thrombosis, DVT, hyperglycaemia, neuropathy) Post-surgical complications in patients who completed all four cycles of NAC then underwent inguinal LND (n = 22) were fewer or comparable to contemporary LND experience with/without chemo. at same institution [55]
Pizzocaro et al. (2009) [29]	Retrospective case series	NAC, n = 6 No comparator	Median age: 54 yrs (IQR 48.4–64.3) SCC in all Patients had fixed or relapsed nodal metastases T stage at first treatment: T1 1/6 (16.7%), T2 2/6 (33.3%), T3 2/6 (33.3%), Tx 1/6 (16.7%) N stage at NAC: N2 1/6 (16.7%), N3 5/6 (83.3%) Tumour grade: G1 1/6 (16.7%), G2 2/6 (33.3%), G3 3/6 (50%)	Paclitaxel, cisplatin, 5-FU (three weekly cycles) Median number of cycles: two (range 2–7) Response evaluated after two and four cycles	Not reported	ORR (RECIST criteria) OS Toxicity	ORR = 83.3%; CR = 4/6 (66.7%); PR = 1/6 (16.7%); no response = 1/6 (16.7%) Median OS = 18.0 months Two patients with pathologic CR who completed four cycles achieved long-term remission (>2 yrs) One patient with PR who received two cycles and underwent surgery also achieved long-term survival (46 months) One non-responder progressed and died within 4 months Two achieved clinical CR after two cycles but did not complete therapy and relapsed quickly (10 and 4 months); both died of penile cancer Completing four cycles is important in all responders, and post-chemotherapy surgical resection is essential in all responders Toxicities not systematically reported: severe nausea and vomiting (n = 1), haematologic toxicities did not exceed Grade 2
Theodore et al. (2008) [28] EORTC protocol 30992	Phase II prospective, single-arm trial	NAC, n = 7 No comparator Advanced disease (T4/N3/M1), n = 21	Median age: 55 yrs (range 36–77) SCC in all patients Inclusion criteria: T3-4, N1-3 or M1, with ≥1 lesion ≥ 20 mm on CT WHO PS: 0 = 12/28 (42.9%), 1 = 13/28 (46.3%), 2 = 3/28 (10.7%) T3 and/or N1 and/or N2 (planned for NAC prior to radical surgery) 7/28 (25%) Advanced disease: T4 or N3 8/28 (28.6%) M1 locally invasive 3/28 (10.7%) M1 distant 10/28 (35.7%) Two not eligible for ORR assessment: 1 >75 yrs old (exclusion criterion), one undiagnosed sarcoidosis of mediastinal lymph nodes instead of metastatic cancer	Cisplatin, irinotecan (28-day cycle) NAC (T3 or N1-N2): max. four cycles before surgery Advanced (T4 or N3 or M1): up to eight cycles Median number of cycles: four (range 3–4 for NAC, 1–8 for advanced) Dose modifications 1/7 NAC, 4/21 advanced Dose withheld 4/7 NAC, 9/21 advanced Treatment stopped early 0 NAC, 4/21 advanced	Not reported	ORR (RECIST criteria) CTCAE toxicity	A total of 26 eligible patients evaluable for response, ORR = 30.8% (95% CI 14.3–51.8%); CR = 2/26 (NAC = 1/7 [14.3%]); PR = 6/26 (NAC = 1/7 [14.3%]); SD = 8/26 (NAC = 4/7 [57.1%]); PD = 10/26 (NAC = 1/7 [14.1%]) Post-NAC pathologic CR on LND in 3/7 (42.9%) Toxicity acceptable, Grade 3 diarrhoea in 3/28 (10.7%), Grade 4 neutropenic fever in 2/28 (7.1%), Grade 3–4 anaemias 5/28 (17.9%), Grade 3–4 fatigue 3/28 (10.7%) Powered to exclude minimal clinically important response of 30%, lower bound of CI being 14.3% indicates study was negative but pathologic CR post-NAC warranted further investigation
Leijte et al. (2007) [27]	Retrospective cohort study	NAC, n = 20 No comparator	Mean age: 62 yrs (range 35–79) SCC in all (including 1 sarcomatoid variant) Indication for NAC: irresectable locally/regionally advanced M0 disease, including fixed inguinal nodes or irresectable locally advanced disease T stage: T1 1/20 (5%), T2 4/20 (20%), T3 7/20 (35%), T4 8/20 (40%) N stage: N0 2/20 (10%), N1 1/20 (5%), N3 17/20 (85%) Tumour differentiation: good 8/20, moderate 5/20, poor 6/20, unknown 1/20	Bleomycin (n = 3), bleomycin/vincristine/methotrexate (n = 5), 5-FU/cisplatin (n = 1), bleomycin/cisplatin/methotrexate (n = 10), cisplatin/irinotecan (n = 1) Treatment stopped if progression or severe toxicity	23	OS ORR (RECIST criteria) CTCAE toxicity Subsequent surgery rate	Evaluable for response, n = 19 (one died before response assessment); ORR = 12/19 (63%); CR = 2/19 (10.5%); PR = 10/19 (52.6%); SD/PD = 7/19 (37%) Overall 5-yr OS 32% (95% CI 17–62%) for whole cohort; significantly longer OS in NAC-responders (5-yr OS 56%, 95% CI 34–94%) as compared with non-responders (all patients died within 9 months, log-rank test *p* = 0.012) Severe toxicity in 4/20 (20%), three of these four received bleo/cis/MTX; three toxic deaths; treatment discontinuation from toxicity in 1/20; Grade 1 and 2 nausea and fatigue almost universally reported; Grade 1 or 2 alopecia in large number of patients Overall, 9/12 responders underwent curative-intent surgery → 2/9 pT0N0 and 8/9 long-term survivors with NED Overall, 3/7 non-responders underwent surgery for local control → all died from recurrent disease within 8 months of surgery NAC valuable in otherwise incurable locally advanced/node-positive disease but surgery should only be performed in responders
Bermejo et al. (2007) [26]	Retrospective case series	NAC, n = 10	Advanced penile cancer with inguinal and/or pelvic LN involvement who received surgical consolidation after NAC	Combination chemotherapy, paclitaxel/cisplatin/ifosfamide n = 3/10 (others not reported in available abstract)	Not reported	ORR (RECIST criteria) Pathological N status OS Surgical morbidity	ORR = 50%; CR = 4/10 (40%); PR = 1/10 (10%); SD = 5/10 (50%) pN0 = 3/10 (30%), all received paclitaxel/cis-platin/ifosfamide Complications: three major (bleeding in 1/10; acute renal failure in 1/10; DVT in 1/10) and four minor (skin breakdown in 3/10; seroma in 1/10) For ≤3 pathologic LNs post-surgery, n = 7 → NED in four, and three died at last follow-up For >3 pathologic LNs post-surgery, n = 3 → all died Median OS 26 months, 5-yr OS 40% Median OS by nodal burden: 48 months (≤3 nodes) vs. 23 months (>3 nodes), *p* = 0.116

Abbreviations: AC = adjuvant chemotherapy. BMP = bleomycin, methotrexate, cisplatin. CI = confidence interval. CR = complete response. CTCAE = Common Terminology Criteria for Adverse Events. CSS = cancer-specific survival. DSS = disease-specific survival. DFS = disease-free survival. ECOG PS = Eastern Cooperative Oncology Group Performance Status. ENE = extranodal extension. 5-FU = 5-flurouracil. HR = hazard ratio. IQR = interquartile range. LN = lymph node. (P)LND = (pelvic) lymph node dissection. NAC = neoadjuvant chemotherapy. NED = no evidence of disease. ORR = objective response rate. OM = overall mortality. OS = overall survival. PD = progressive disease. PFS = progression-free survival. PR = partial response. QoL = quality of life. SCC = squamous cell carcinoma. SD = stable disease. WHO = World Health Organisation.

##### Efficacy Outcomes of Neoadjuvant Chemotherapy for Penile Cancer

Amongst studies reporting radiological response to neoadjuvant chemotherapy, ORR defined by RECIST radiologic criteria varied substantially, ranging from approximately 29% to 90%, with a median ORR of 50% across the 21 studies that reported this outcome. Lower ORRs (approximately 29–40%) were most observed in cohorts using non-TIP or heterogeneous platinum-based regimens. In contrast, higher ORRs (≥60–85%) were reported in studies employing multi-agent taxane- and platinum-based combinations (including TIP, TPF, and intra-arterial regimens). High ORRs were observed in both very advanced nodal disease cohorts (including cN3-predominant series) and in cohorts with less extensive nodal involvement, indicating that response rates were not solely driven by disease stage.

Pathological response outcomes were reported heterogeneously, most commonly as pathological complete response (pCR) or pathologic nodal complete response (ypN0) following consolidative surgery. Reproducible estimates of pCR/ypN0 clustered between ~10% and 25%, including ~10% in a prospective phase II TIP trial and ~15–25% in larger retrospective series using taxane-based NAC. Several smaller series reported higher apparent pCR/ypN0 proportions, including 30–43% in highly selected surgical consolidation series or very small NAC subgroups, reflecting denominator effects and selection for surgery among responders. Interpretation of pathological response outcomes is limited by substantial heterogeneity in assessment and reporting across studies. Reported pCR/ypN0 rates are influenced by differences in analytic denominators, with many series reporting pathological outcomes only in patients proceeding to consolidative surgery, thereby introducing selection bias toward responders. Additionally, variation exists in pathological definitions (e.g., pCR vs. nodal clearance/ypN0), baseline nodal stage distribution (cN2 vs. cN3 cohorts), and treatment regimens and durations. The absence of consistent intention-to-treat reporting further limits comparability and may result in overestimation of true pathological response rates.

Median progression-free survival (PFS) was reported in nine studies and ranged from ~4 to 26 months, with a median of reported median PFS values of approximately 11 months. Median overall survival (OS) was reported in 13 studies, ranging from ~10 to 75 months, with a median of reported median OS values of approximately 18 months. Longer survival was consistently observed in patients achieving radiological response, pathological downstaging, or ypN0 status and in those able to undergo complete consolidative lymphadenectomy, while poor outcomes were associated with progressive disease during NAC and high residual nodal burden.

##### Neoadjuvant Versus Adjuvant Chemotherapy for Penile Cancer

Comparative data evaluating NAC versus adjuvant chemotherapy (AC) were reported in a subset of retrospective cohort studies. No analysis demonstrated superior oncological outcomes with NAC compared with AC. Several studies reported outcomes favouring AC [33,37,49,52,54]. Noronha et al. (2024) observed longer overall survival in patients treated with AC compared with NAC (median OS 89.9 vs. 14.4 months) [49]. Soares et al. (2024) similarly reported longer median progression-free survival (11 vs. 4 months) and overall survival (49 vs. 21 months) with AC relative to NAC [52]. Nicolai et al. (2016) demonstrated longer progression-free survival and higher two-year disease-free survival rates in AC-treated patients compared to an NAC cohort [37]. Giannatempo et al. (2014) reported a higher frequency of durable remission in patients receiving AC, and AC was associated with improved survival outcomes on multivariable analysis [33]. You et al. (2025) reported inferior progression-free survival in an NAC group compared with AC, while overall survival did not differ significantly between groups [54]. Other studies reported no significant difference in oncological outcomes between NAC and AC [40,53]. Dhasthakeer et al. (2025) found similar disease-free survival between NAC and AC across multiple chemotherapy regimens [53]. Necchi et al. (2017) reported differences in recurrence-free survival between treatment timing groups that numerically favoured AC, while overall survival did not differ significantly between NAC and AC [40].

##### Toxicities of Neoadjuvant Chemotherapy for Penile Cancer

Toxicity reporting across NAC studies was heterogeneous, but available data suggest that treatment-related adverse effects were common and varied by regimen. In the pivotal phase II TIP study by Pagliaro et al. (2010), toxicity was considered manageable, with Grade 3 infection the most frequent severe adverse event (n = 5/30), and each major haematological complication of anaemia, thrombocytopenia and neutropenia occurring once [30]. There were no Grade 5 treatment-related events reported in this trial and there were fewer or comparable rates of post-surgical complications in those who completed all four cycles of NAC then underwent ILD, as compared with the contemporary LND experience without chemotherapy at the same institution. By contrast, prospective TPF studies demonstrated substantially greater toxicity burdens. In the CRUK/09/001 phase II study, 65.5% of patients experienced at least one Grade 3 or 4 adverse event. Larger retrospective taxane-based series generally suggest more acceptable tolerability in contemporary practice, with one international multicentre real-world study reporting Grade ≥ 3 treatment-related toxicity in 17% and no treatment-related mortality [31]. Across the neoadjuvant chemotherapy studies in penile cancer, the most consistently reported severe toxicities were haematological, including neutropenia, febrile neutropenia, anaemia, and infection, while non-haematological toxicity included gastrointestinal adverse effects, neuropathy, and renal toxicity. These findings suggest that although taxane–platinum neoadjuvant regimens are feasible in selected patients, toxicity remains clinically important and may influence both completion of systemic therapy and subsequent fitness for consolidative surgery.

#### 3.2.2. Neoadjuvant Radiotherapy for Penile Cancer

Five studies evaluated neoadjuvant radiotherapy, including one contemporary prospective chemoradiotherapy trial and several retrospective series (Table 2). An early historical study using radiotherapy alone with neoadjuvant intent reported low response rates and poor long-term survival, limiting contemporary relevance [21]. A more recent prospective single-arm clinical trial employing modern radiotherapy techniques with concurrent chemotherapy demonstrated high metabolic and radiologic response rates, with approximately 70% achieving objective response on PET-based assessment [22]. Over half of patients subsequently underwent salvage or consolidative surgery. One- and two-year OS rates in this cohort were approximately 75% and 45%, respectively, despite a predominance of cN3 disease (88% of patients). Comparative retrospective analyses suggested similar disease control between neoadjuvant chemoradiotherapy and NAC in selected high-risk populations, though sample sizes were small and confidence intervals wide [48,56].

#### 3.2.3. Neoadjuvant Immunotherapy for Penile Cancer

Five studies addressed immunotherapy with neoadjuvant intent or perioperative relevance, including one phase II trial, two ongoing neoadjuvant protocols, and limited basket-trial or real-world data demonstrating surgical consolidation following response (Table 3) [57,58,59,60,61]. The most robust evidence came from a phase II combination regimen incorporating PD-1 blockade with chemotherapy and EGFR-targeted therapy, which achieved ORR > 80% and pCR approaching 50%, with durable disease control observed at extended follow-up. Toxicity was substantial but manageable, with Grade ≥ 3 adverse events in approximately 40%. Ongoing trials are evaluating chemoimmunotherapy approaches with pCR as the primary endpoint and structured post-treatment surveillance. Small basket-trial series demonstrated durable responses in biomarker-selected patients, including successful surgical consolidation following immunotherapy, though numbers remain extremely limited.

Toxicity data for neoadjuvant immunotherapy in penile SCC remain limited but suggest that combination regimens may carry appreciable treatment-related morbidity. In the mature phase II study of toripalimab, nimotuzumab, and taxane-based chemotherapy, Grade 3–4 treatment-related adverse events occurred in 41.4% of patients, with neutropenia, anaemia, febrile neutropenia, and rash among the most common severe events, but no treatment-related deaths were reported [61]. The most common potentially immune-related adverse events were all Grade 1 or 2, including hypothyroidism (n = 5/29), hyperthyroidism (n = 3/29), and adrenal insufficiency (n = 2/29). Dose reduction due to Grade 3 or 4 toxicity occurred in 3/29, and 1/29 discontinued due to Grade 3 asthenia. The available neoadjuvant immunotherapy literature therefore suggests that the toxicity profile is driven largely by the cytotoxic chemotherapy backbone rather than unexpected immune-mediated toxicity alone, although immune-related adverse events remain relevant and are likely to become more important as checkpoint inhibitor-based strategies are expanded into broader perioperative use. Given the requirement to preserve surgical candidacy in this setting, consistent reporting of CTCAE toxicity, treatment delays, dose reductions, and failure to proceed to surgery will be particularly important in future neoadjuvant immunotherapy studies.

#### 3.2.4. Neoadjuvant Molecularly Targeted Systemic Therapy for Penile Cancer

Three studies evaluated molecularly targeted systemic therapy with potential neoadjuvant application (Table 4) [62,63,64]. These included one EGFR-targeted retrospective cohort, one prospective phase II pan-HER tyrosine kinase inhibitor trial, and one protocol for a trial of VEGFR/MET-targeted therapy. Targeted monotherapy produced modest ORRs (25–35%), with short median PFS (~2–4 months) and median OS generally under 14 months in unselected populations. However, a consistent finding across studies was that responders who proceeded to consolidative surgery experienced substantially longer survival, including occasional long-term survival up to 3–4 years at last follow-up. No study demonstrated meaningful pCR with targeted monotherapy alone. One clinical trial currently underway incorporates the preoperative window to test targeted agents as induction therapy, with response-adapted surgery forming a central component of the treatment strategy [63].

Toxicity data for molecularly targeted systemic therapy were more limited, but available studies suggest lower rates of severe toxicity than multi-agent cytotoxic chemotherapy, albeit alongside lower efficacy. In the phase II dacomitinib study, Grade 3 adverse events were observed in 10.7% of patients and were limited primarily to skin rash, with no treatment-related deaths reported [64]. In retrospective EGFR-targeted series, rash was the most frequent adverse event and was usually low-grade, while serious events such as cellulitis, thrombocytopenia, or bronchospasm were uncommon [62]. Overall, the tolerability profile of targeted monotherapy appears more favourable than that of taxane–platinum regimens, but this advantage must be interpreted alongside the modest response rates and lack of reproducible pathological complete responses reported to date.

## 4. Discussion

### 4.1. Contextualisation of Current Guidelines on Neoadjuvant Therapy for Penile Cancer and the Evidence–Implementation Gap

International guidelines from EAU-ASCO, French AFU Cancer Committee, NCCN, and ESMO-EURACAN consistently position neoadjuvant cisplatin- and taxane-based combination chemotherapy as the preferred neoadjuvant strategy for patients with high-risk, clinically node-positive penile cancer, particularly those with cN2-3 disease, in whom upfront surgery alone is unlikely to be curative (Table 5) [6,9,10,11,65,66]. NAC prior to lymphadenectomy is intended to provide early treatment of systemic disease and down-size tumour burden to improve resectability. A prior systematic review demonstrated appreciable mean 5-year survival of 56.9% in a favourable subgroup of patients who responded to NAC and proceeded with consolidative surgical resection [67]. By contrast, oncological outcomes were consistently found to be considerably poorer in non-responding patients across NAC studies (Table 1), reflecting caution in guidelines regarding the role of surgery in these poor-prognosis patients. Recommended NAC regimens centre on paclitaxel, cisplatin, and ifosfamide (TIP) and docetaxel, cisplatin, and 5-FU (TPF), based primarily on phase II trial evidence. A phase II trial of patients with cN2-3 penile cancer treated with TIP demonstrated a 50% ORR, median time to progression of 8.1 months, median OS 17.1 months, and long-term DFS in 67% of patients who responded, as compared with 7% following no response to NAC [30]. The phase II trial evaluating TPF failed to meet its primary endpoint but reported a 38.5% ORR in a mixed cohort of both locally advanced and metastatic penile cancer [31]. Two-year PFS and DSS rates were 12% and 28% in a further prospective study of neoadjuvant TPF that did not include metastatic disease [35]. Toxicity from TPF was significant in both studies, with 65.5% of patients experiencing Grade ≥ 3 adverse events in the trial by Nicholson et al. and 23% of patients discontinuing treatment due to toxicity in the study by Djajadiningrat et al. Taken together, recommendations for NAC in penile cancer with clinically involved nodes reflects expert consensus informed by limited phase II clinical trial data and pooled retrospective series, with a lack of randomised controlled trial data indicative of the rarity of the disease. As such, guidelines uniformly acknowledge the low strength of evidence underpinning these recommendations and highlight uncertainty around the selection of patients most likely to benefit from preoperative chemotherapy. A retrospective analysis in a large, multicentre cohort study demonstrated the greatest benefit from NAC versus upfront lymphadenectomy in the cN3 subgroup, but bilateral inguinal lymph node disease was also a significant negative prognostic factor, suggesting the potential importance of multimodal therapy in this cohort [43].

Across retrospective series, ORR following NAC is variable but broadly aligns with prospective data, ranging between approximately 30% and 90% in the included studies. Lower response rates were observed in smaller or less selected cohorts and less intensive chemotherapy regimens, such as those in Soares et al. (2024) [52], which reported an ORR of 29% with no complete responses in a cohort treated with doublet platinum-based NAC, and Nicolai et al. (2016) [37], which demonstrated an ORR of 42.9% with a CR rate of only 3.6%, with all patients receiving less than the recommended four cycles of NAC. In contrast, higher response rates were observed in larger or more contemporary cohorts, including those in Chahoud et al. (2022) (ORR 56%, CR 7.6%) and Rose et al. (2024) (ORR 57.2%, CR 13.9%) [47,51]. Earlier series, such as Dickstein et al. (2016) [36], reported ORRs as high as 65%, with pathological nodal complete response rates of 16.4%, with most patients receiving TIP (n = 54/61). The highest retrospective ORR of 89.5% was reported by Dhasthakeer et al. (2025), despite a doublet regimen of 5-fluorouracil and cisplatin being used in the majority of patients (n = 28/38), potentially reflecting the relatively high number of cycles completed (given as six cycles) [53]. Clinicopathological characteristics of NAC-treated patients were not reported in this study to provide further insight into possible biological rationale for the relatively high ORR observed in this cohort.

Multiple studies demonstrate that response to NAC is a key determinant of oncological outcomes. In the large multicentre cohort by Rose et al. (2024) [51], responders had a median OS of 73 months compared to 17 months in non-responders (confidence intervals not reported), with a corresponding median PFS of 50 versus 11 months (*p* < 0.01). Similarly, Xu et al. (2019) demonstrated a median OS of 54 months in responders compared to 15 months in non-responders (*p* < 0.001) [44], and Dickstein et al. (2016) reported 5-year OS rates of 50.1% in responders compared to 7.7% in patients with progressive disease [36]. These findings are consistent with prospective data from Pagliaro et al. (2010), in which response to NAC was strongly associated with improved PFS and OS [30]. Another consistent finding across studies is the importance of achieving disease control to enable consolidative surgery. Rates of conversion to resectability following NAC are substantial in responders, with Dickstein et al. (2016) [36] reporting that 85% of patients proceeded to surgery following NAC, and Pagliaro et al. (2010) [30] reporting a consolidative surgery rate of 73.3%. In contrast, patients with progressive disease during NAC have extremely poor outcomes, as demonstrated by a median OS of 10.9 months in patients with progressive disease in the Dickstein et al. cohort.

Despite these prognostic associations with NAC response, several retrospective comparative analyses have failed to demonstrate a clear overall survival benefit for NAC in unselected populations. Necchi et al. (2019) found that NAC was not associated with improved OS (HR for death 1.61, 95% CI 1.04–2.47, *p* = 0.031) or RFS (HR for recurrence 2.00, 95% CI 1.31–3.07, *p* = 0.001) compared to no perioperative therapy on multivariable analysis, although the majority of patients (~60%) did not have cN3 disease for which NAC is currently recommended. Indeed, a subgroup analysis of this cohort found that while NAC was ineffective on Kaplan–Meyer analyses for OS in patients with cN0 and cN1-2 disease, a non-statistically significant OS improvement was observed in cN3 patients treated with NAC compared to no perioperative treatment (*p* = 0.15, median OS not reported) [43]. Chipollini et al. (2018) similarly reported no significant association between NAC and OS, CSS, or PFS (multivariable HR NAC vs. LND-alone: PFS HR 1.52 (*p* = 1.04); CSS HR 1.59 (*p* = 0.091); OS HR 1.52 (*p* = 0.11)), although there were significant prognostically relevant baseline differences between the LND-only and NAC groups, which could have diluted any treatment effect from NAC as most pN2/N3 patients received systemic therapy (67%) whilst most pN1 patients had LND alone (65%) [42]. Reddy et al. (2017) found that NAC was associated with worse RFS on univariate analysis compared to AC (HR 2.90, 95% CI 1.76–4.74, *p* < 0.001); however, there was likewise a significant risk of selection bias from baseline differences in underlying disease severity, with NAC use being strongly associated with more advanced nodal disease (50% of NAC patients had cN3, Cochran–Armitage trend test vs. AC, *p* < 0.001) [41].

Furthermore, subgroup analyses suggest that NAC may confer benefit in patients with the highest disease burden. Bandini et al. (2020) demonstrated a statistically significant 23% improvement in 2-year OS in “NAC-eligible” patients with cN2 disease and pelvic nodal involvement or cN3 disease (*p* = 0.002), with NAC independently associated with lower overall mortality in this subgroup (HR 0.28; 95% CI 0.13–0.62, *p* = 0.002) [45]. Similarly, Zargar-Shoshtari et al. (2016) reported significant improvements in OS (HR 0.10; 95% CI 0.02–0.44, *p* = 0.002) and CSS (HR 0.15; 95% CI 0.03–0.70, *p* = 0.02) with NAC compared to no perioperative therapy in patients with advanced nodal disease (cN3 in all patients) [38]. These findings support current guideline recommendations favouring NAC in patients with bulky or fixed nodal disease.

Toxicity remains a significant limitation of current NAC regimens. Across both prospective and retrospective studies, Grade ≥ 3 toxicities, predominantly haematological, occurred frequently with a range across included studies of 17–65%. Moreover, treatment discontinuation rates of up to approximately 25% reported were in some cohorts. Although treatment-related mortality is rare, these toxicity profiles are clinically relevant and must be taken into consideration in treatment planning, given data indicating the oncological importance of preserving patients’ capacities to proceed with surgical consolidation.

Despite international guideline endorsement, real-world uptake of NAC appears inconsistent and frequently lower than might be expected based on guideline positioning. European data from the E-PROPS survey demonstrated very low adherence to EAU systemic treatment recommendations, including neoadjuvant strategies, with the authors highlighting uncertainty as to whether this reflected limited clinician familiarity, institutional constraints, or the inherently weak evidence base supporting these recommendations [68]. Similar patterns have been observed in Australia, where a small retrospective study reported no use of neoadjuvant chemotherapy in men with penile cancer even in high-risk or clinically node-positive cases [69]. More contemporary Australian single-centre experience from a high-volume institution suggests greater alignment with guideline-recommended multimodal care, indicating that access to subspecialist expertise and multidisciplinary pathways may be critical determinants of neoadjuvant utilisation [46]. Reports from high-volume North American centres and registry-linked institutional series suggest higher rates of NAC use [70,71], with some cohorts reporting use of NAC in up to 26% of node-positive patients [72]. Broader international survey data, including those from the Global Society of Rare Genitourinary Tumours, which incorporates Asian centres, further corroborate marked geographic and institutional variability in systemic treatment sequencing [73]. Collectively, these observations suggest that limited real-world penetration of neoadjuvant chemotherapy for penile cancer is likely multifactorial and may reflect variability in clinician experience, logistical barriers to delivering intensive chemotherapy, concerns regarding toxicity in often comorbid patient populations, and persistent uncertainty arising from the absence of definitive comparative trials.

### 4.2. Comparison of Neoadjuvant Versus Adjuvant Chemotherapy for Penile Cancer

Among studies directly comparing perioperative treatment timing, AC was more frequently associated with longer survival or disease control outcomes, while NAC was not associated with superior outcomes in any comparative analysis [33,37,40,49,52,53,54]. The interpretation of these findings must be considered within the context of substantial confounding by indication and pathway effects. By design, NAC is preferentially selected for patients with bulky, fixed, or clinically unresectable nodal disease with poor baseline prognosis, whereas AC cohorts are often enriched for patients who were considered operable up-front, successfully underwent complete resection, and survived long enough postoperatively to receive systemic therapy, factors that are not accounted for in retrospective study designs. These systemic baseline differences create selection bias that may obscure the relative benefit of NAC within its intended patient population. Furthermore, retrospective treatment-timing comparisons are vulnerable to time-related biases and informative censoring. Immortal time and lag-time effects can be introduced depending on how the index date is defined (e.g., first chemotherapy dose versus surgery) and whether patients who deteriorate on NAC and never reach surgery are excluded from analytic cohorts. These issues are well-recognised in perioperative oncology comparisons and are challenging to correct without prospective, randomised allocation.

### 4.3. Limited Evidence for Neoadjuvant Radiotherapy in Penile Cancer

Radiotherapy has a strong conceptual precedent in other SCCs, where definitive or perioperative chemoradiation is central to curative care [17,18,19,20]. A single retrospective study evaluated preoperative radiotherapy in 12 patients with cN3 penile cancer, reporting an objective response in only two (17%) patients, with a 5-year OS rate of just 17% [21]. These data are not generalisable to current clinical practice as the study reported on historical radiotherapeutic techniques that are not directly comparable to modern radiotherapy. Contemporary neoadjuvant or induction chemoradiotherapy series suggest the approach can achieve meaningful metabolic and radiologic responses, particularly in selected high-risk cN3-predominant populations, and may facilitate salvage or consolidative surgery in a subset [22,48,56]. However, the evidence overall for this efficacy is weak, with only one small prospective trial demonstrating response to preoperative chemoradiotherapy [22], and otherwise small cohort numbers, selection bias, and inconsistent reporting of late toxicity. As such, guidelines suggest reserving neoadjuvant chemoradiotherapy for patients requiring treatment for involved lymph nodes whilst awaiting surgery to improve resectability, who are unfit for conventional multi-agent chemotherapy [10]. The randomised controlled InPACT trial for penile cancer includes a neoadjuvant chemoradiotherapy arm for comparison against multi-agent NAC, the results of which are eagerly anticipated [8,56].

### 4.4. Emerging Evidence for Neoadjuvant Immunotherapy in Penile Cancer

The rationale for immunotherapy in penile SCC is supported by convergent observations across the translational and clinical literature. The penile tumour microenvironment contains prognostically relevant immune infiltrates, and PD-L1 expression is reported in a substantial proportion of tumours (up to 79% in some series) [74,75,76,77], with PD-L1 expression being predictive of immunotherapy efficacy [75,78,79], thus supporting the biological plausibility of PD-1/PD-L1 blockade. Nonetheless, immunotherapy monotherapy activity in advanced penile SCC has generally been modest and inconsistent, with use largely restricted to relapsed or metastatic contexts in clinical trial settings [15]. These observations mirror other squamous cell malignancies where combination strategies, involving immunotherapy with either chemotherapy or radiotherapy, have outperformed immunotherapy monotherapy. Emerging data on neoadjuvant combination approaches, particularly chemoimmunotherapy with or without molecularly targeted agents, are now reporting higher short-term efficacy signals than historical chemotherapy alone, including markedly higher pCR rates in one phase II experience [61]. Whilst these results are encouraging, cautious interpretation is required in the absence of randomised controlled trial comparisons between immunotherapeutic regimens and the current standard-of-care NAC. High pCR rates in small, single-arm studies can reflect patient selection, stage mix, and the requirement to proceed to surgery for pathological assessment without intention-to-treat analysis. Moreover, intensified regimens may trade efficacy for toxicity, and CTCAE Grade ≥ 3 adverse event rates remain an important consideration when attempting to deliver therapy preoperatively without compromising surgical candidacy. Nevertheless, immunotherapy is now a leading candidate to shift neoadjuvant efficacy in penile cancer, pending replication of results across independent cohorts in further prospective clinical trials [57,58,60].

### 4.5. Limited Evidence for Neoadjuvant Molecularly Targeted Systemic Therapy in Penile Cancer

Molecularly targeted systemic therapy alone lacks clinical trial and observational evidence for neoadjuvant application in penile SCC. Evidence from studies of advanced or metastatic disease that incorporated consolidative surgery demonstrated only modest ORRs (~20–35%) and short median PFS (~2–4 months) with EGFR-directed monoclonal antibodies and pan-HER tyrosine kinase inhibitors in largely unselected populations [62,64]. In a prospective phase II study of dacomitinib in chemotherapy-naïve advanced penile SCC, the ORR was 32.1% with a median PFS of 4.1 months and median OS of 13.7 months, without evidence of frequent or complete pathological responses [64]. Similarly, a retrospective study with cetuximab or panitumumab reported limited durable responses and no reproducible signal of meaningful pathological complete response with targeted monotherapy [62]. The available evidence thus does not demonstrate a predominant role for novel molecularly targeted agents alone in the curative-intent neoadjuvant setting; however, several biological considerations may justify continued investigation. Penile SCC shares molecular and genomic features with other squamous cell malignancies, particularly head and neck SCC, including recurrent alterations in TP53, PI3K/AKT/mTOR pathway dysregulation, Notch pathway mutations, receptor tyrosine kinase signalling abnormalities, and HPV-associated APBOEC mutational signatures [80,81,82,83]. Single-cell and genomic profiling studies have further demonstrated substantial molecular heterogeneity across HPV-positive and HPV-negative tumours, identifying potentially targetable alterations in RTK-RAS, DNA damage repair, and cell cycle pathways [81,82,83]. Moreover, the therapeutic value of molecularly targeted agents in penile SCC may lie in rational combination approaches, given preclinical studies and data from other malignancies suggesting that EGFR inhibition can enhance radiosensitivity and potentiate chemotherapy efficacy [84,85,86,87], while pathway-directed agents may augment responsiveness to immune checkpoint blockage through tumour microenvironment modulation [13,14,80,88]. In this context, further research could investigate targeted agents as sensitising or synergistic components within multimodal regimens, such as enhancing cisplatin–taxane-based cytotoxic combinations or supporting induction chemoimmunotherapy or chemoradiotherapy for chemotherapy-ineligible patients to facilitate response-adapted surgical consolidation.

### 4.6. Comparison of Toxicities of Systemic Neoadjuvant Therapies for Penile Cancer

Across systemic neoadjuvant modalities, toxicity appears to track more closely with regimen intensity than with treatment class alone. Taxane–platinum chemotherapy remains the best-supported approach, but it is associated with clinically meaningful haematological and infectious toxicity, particularly with more intensive triplet regimens. Notably, TPF appears less well tolerated than TIP in prospective studies. Early neoadjuvant immunotherapy combinations have reported promising efficacy signals, but the currently available data suggest that severe toxicity remains substantial, again largely reflecting the accompanying chemotherapy backbone rather than isolated immune-related events. By contrast, targeted monotherapy appears better tolerated, with lower rates of Grade ≥ 3 toxicity and rash as the dominant adverse effect, but this relative tolerability is offset by modest efficacy and limited evidence of deep pathological response. Taken together, these data reinforce that treatment selection in clinically node-positive penile cancer must balance oncological ambition against the need to preserve surgical fitness, particularly in patients with bulky groin disease, baseline comorbidity, or impaired performance status. In addition to astute reporting of CTCAE toxicity, future neoadjuvant studies should therefore also report treatment completion, dose intensity, delays to lymphadenectomy, and rates of failure to proceed to surgery, as these are likely to be more clinically informative than toxicity counts alone in this setting.

### 4.7. Predictive Biomarkers and Patient Selection for Neoadjuvant Systemic Therapy

There are emerging opportunities to biologically stratify neoadjuvant treatment and patient selection based on novel predictive biomarkers. Penile SCC is increasingly being recognised as having dual pathogenesis: HPV-associated tumours driven by viral oncogenes (E6/E7) and HPV-independent tumours often associated with chronic inflammation (e.g., lichen sclerosis, phimosis) and characterised by different somatic tumour suppressor gene alterations and growth factor signalling dependence [89,90,91,92]. This aetiological divide may have therapeutic implications as HPV status is associated with distinct, molecular signalling profiles that may be targetable or predictive of treatment response.

As in other HPV-driven squamous cell malignancies, preliminary evidence indicates increased sensitivity to chemotherapy and improved responses to immunotherapy and chemoradiotherapy for HPV-positive penile SCC as compared with HPV-negative disease [7,93,94,95,96,97]. A recent meta-analysis investigated the prognostic significance of both HPV DNA tumour positivity and p16-positive immunohistochemistry as a surrogate of HPV-driven carcinogenesis [98]. Prolonged cancer-specific survival (HR  =  0.40, 95% CI [0.20, 0.80], *p*  =  0.009) and OS (HR  =  0.49, 95% CI [0.29, 0.85], *p*  =  0.01) were associated with p16 positivity, whilst improved cancer-specific survival (HR  =  0.54, 95% CI [0.35, 0.83], *p*  =  0.005) but not OS (HR  =  0.86 95% CI [0.70, 1.07], *p*  =  0.17) was associated with HPV DNA positivity. The HERCULES (LACOG 0218), non-randomised, phase II trial evaluated pembrolizumab plus platinum/5-FU chemotherapy as a first-line therapy for advanced penile SCC and found that HPV-status was a predictive biomarker for immune checkpoint inhibition, with HPV-positive patients achieving significantly higher ORRs (55.6% [95% CI, 21.2–86.3%] vs. 35% [95% CI, 15.4–59.2%]) and longer PFS (7.6 [95% CI, 0.7–not estimated] months vs. 5.6 [95% CI, 1.3–7.3 months]) compared with HPV-negative patients [99]. The proposed mechanism for these differences is that HPV-positive tumours often exhibit higher tumour-infiltrating lymphocyte density and an inflammatory microenvironment, with the expectation of more immune-mediated responses in this cohort [99,100,101]. Recent molecular studies of advanced penile SCC have demonstrated that HPV-negative tumours more frequently express phosphorylated EGFR compared with HPV-positive tumours [91,102], possibly because HPV viral oncoproteins E6/E7 bypass the need for traditional growth factor signalling, supporting the hypothesis that HPV-negative disease may be more sensitive to EGFR-directed strategies such as cetuximab or panitumumab [102].

Beyond HPV, several other candidate biomarkers warrant discussion. PD-L1 expression is common in penile SCC and provides a biological rationale for immunotherapy [100], but its predictive value remains inconsistent. In the HERCULES trial, PD-L1 expression was not associated with treatment response despite a high prevalence of positivity [99]. Tumour mutational burden (TMB) may be more informative in selected patients: in a contemporary molecular analysis, high TMB was associated with strong PD-L1 expression, HPV negativity, and worse overall survival, while exploratory HERCULES data suggested improved ORR and PFS in TMB-high tumours [99,103,104]. Genomic profiling studies have also identified recurrent alterations in TP53, NOTCH, PI3K/AKT/mTOR, RTK-RAS, and DNA damage response pathways, reinforcing the biological heterogeneity in penile SCC and the possibility of future biomarker-enriched neoadjuvant trials [89,92,100]. At present, however, no molecular marker is sufficiently validated to routinely direct standard neoadjuvant treatment selection, and most remain hypothesis-generating.

### 4.8. Limitations

This review has several important limitations. Although the literature search was systematic, the evidence base was not synthesised by quantitative pooling. Substantial heterogeneity was identified amongst studies, which differed in design, eligibility criteria, baseline stage distribution (including variable definitions of “bulky” or “fixed” nodal disease), neoadjuvant regimens and dosing schedules, response assessment methods (RECIST vs. metabolic imaging vs. pathological endpoints), follow-up duration, reporting of toxicity grading, and the extent and timing of surgical consolidation. As a formal meta-analysis was not performed, summary estimates should be interpreted as descriptive rather than definitive measures of comparative effectiveness. Indeed, the methodology of this review did not prospectively include formal risk of bias assessment using structured instruments due to the narrative synthesis approach and marked heterogeneity in the included studies. Instead, relevant potential biases according to study design are critically appraised concurrently with the interpretation of outcomes. Moreover, most included studies were non-randomised and therefore vulnerable to selection bias, confounding by indication, and time-related biases, limiting causal inference. A further important limitation relates to heterogeneity in outcome assessment. Radiologic response is predominantly reported using RECIST criteria, while a minority of studies utilise metabolic (PET-based) assessment, limiting cross-study comparability. Pathological response endpoints are particularly susceptible to bias, as they are typically restricted to patients who undergo consolidative surgery, thereby enriching for responders and introducing selection bias. Inconsistent definitions of pCR and nodal response, together with variable use of intention-to-treat denominators, further complicate interpretation. These factors likely contribute to the wide variability in the reported pCR/ypN0 rates across studies. Collectively, these limitations reinforce the need for standardised reporting and prospective multicentre trials to better define optimal neoadjuvant strategies in penile SCC.

### 4.9. Future Directions

The next phase of progress in neoadjuvant treatment for penile SCC will be defined by coordinated, prospective, internationally harmonised trials capable of resolving sequencing, modality integration, and patient selection. The International Penile Advanced Cancer Trial (InPACT; NCT02305654) represents a pivotal initiative in this transition. InPACT is a multi-centre, randomised, multi-stage controlled trial that has set out to evaluate the relative benefits and sequencing of surgery, neoadjuvant chemotherapy, and neoadjuvant chemoradiotherapy in clinically node-positive disease, with additional evaluation of pelvic management strategies in high-risk patients [8,56]. InPACT is thus uniquely positioned to address whether neoadjuvant therapy improves outcomes beyond surgery alone, whether chemoradiotherapy adds benefit over chemotherapy, and how best to integrate pelvic treatment, recognising a need to define optimal multimodal sequencing. If successfully completed with sufficient recruitment, InPACT has the potential to recalibrate the strength of guideline recommendations to RCT-defined standards. The need for further prospective data is driving several other trials in neoadjuvant treatment for penile cancer (Table 6). Preliminary data on predictive biomarkers support prospective biomarker-integrated trial designs, particularly those incorporating HPV/p16 status, immune profiling, and selected genomic alterations into perioperative treatment stratification.

## 5. Conclusions

Prospective phase II data underpin current guideline endorsement of neoadjuvant taxane–platinum chemotherapy (most commonly TIP/TPF) for men with bulky, clinically node-positive penile SCC, and the broader evidence base dominated by retrospective cohorts demonstrates clinically meaningful radiologic responses but generally modest and variably reported pCR/ypN0 rates, with durable benefit concentrated in responders who proceed to complete surgical consolidation. However, the strength of evidence remains limited by small sample sizes, heterogeneous regimens and endpoints, and the absence of completed randomised trials. Early neoadjuvant combination immunotherapy efficacy signals appear stronger than historical chemotherapy alone but require prospective validation in controlled perioperative pathways. Prioritising trial enrolment, standardised reporting, and biomarker-informed patient selection may facilitate the translation of response gains into durable survival improvements in this rare malignancy.

## Figures and Tables

**Table 2:** Summary of studies assessing neoadjuvant radiotherapy or primary radiotherapy followed by salvage resection of residual, with/without systemic therapy for penile cancer.

Study	Design	Study Population Size	Disease Characteristics	Treatment Details	Median Follow-Up, Months	Outcome Measures	Results
Cooper et al. (2025) [56], Canter et al. (2019) [8] InPACT; NCT02305654	Randomised controlled trial protocol International expert group convened to achieve consensus on inguino-pelvic radiotherapy contouring, planning, dose, and fractionation for each treatment region based on risk status of nodal basins	Trial still recruiting (target 200 participants)	SCC in all Any T, N1-3, M0 suitable for radical treatment ECOG ≤ 2 Low disease burden (one mobile lymph node, no high-risk features) Intermediate disease burden (two ipsilateral mobile lymph nodes, no high-risk features) High disease burden (bilateral, pelvic, or fixed lymph nodes OR ≥ 3 lymph nodes involved radiologically OR presence of high-risk CT features)	Surgery alone vs. NAC (TIP, 4× 21-day cycles) + surgery vs. neoadjuvant chemoradiotherapy + surgery If received NAC and pathological high-risk, second randomisation to either adjuvant CRT or prophylactic pelvic lymph node dissection + adjuvant CRT CRT = concurrent cisplatin once a week (GFR > 45 mL/min), 25 fractions of radiotherapy (5 days/wk for 5 weeks) delivered by IMRT, VMAT or other rotational platform (6–10 MV photons) Both neoadjuvant and adjuvant nodal radiation start after complete excision of primary tumour with negative margins (if penile margins involved, these are defined as part of clinical target volume pre-pubic fat)	Not yet reported	Primary outcome: OS Secondary outcome: DSS, DFS, freedom from locoregional recurrence and distant metastasis, feasibility, toxicity, surgical complications, QoL	Currently recruiting, outcomes not reported
de Vries et al. (2023) [48]	Retrospective cohort analysis	Neoadjuvant CRT (±surgical resection of residual disease), n = 23 NAC, n = 37 Therapeutic PLND, n = 24 Prophylactic PLND, n = 142	Median age: 65 yrs (IQR 57–71) SCC in all patients pT stage: pT1 13%, pT2 44%, pT3 33%, pT4 4.4% cN stage: cN0 14%, cN1 38%, cN2 18%, cN3 31% Baseline characteristics not stratified by treatment modality	NAC regimens: methotrexate/bleomycin/cisplatin; cisplatin/5-FU/docetaxel; cisplatin/5-FU Neoadjuvant CRT: 33 daily fractions (1.5–1.8 Gy) + mitomycin day 1 + capecitabine on radiation days, residual lesions after chemoradiation on PET/CT were surgically resected Adjuvant radiotherapy given for high-risk features in n = 41 (multiple +LNs or ENE) Imaging performed ≥6 wks post-NAC/chemoradiation	79	Recurrence rate and RFS OS CSS	NAC vs. neoadjuvant CRT HR for recurrence or progression = 0.71 (95% CI 0.37–1.4, *p* = 0.32) Adjuvant radiotherapy did not significantly impact recurrence HR 1.3 (95% CI 0.86–2.0) Whole cohort median RFS 11 months (95% CI 7.7–18), 97% recurrences within 2 years Whole cohort median OS 17 months (95% CI 12–22), 5-year OS 33% Whole cohort median CSS 18 months (95% CI 13–29)
Ottenhof et al. (2023) [22]	Prospective, single-arm, clinical trial	Neoadjuvant CRT, n = 33	Median age: 64 yrs (IQR 54–73) SCC in all WHO PS: 0 19/33 (58%), 1 13/33 (39%), 2 1/33 (3%) Locally advanced penile cancer: (i) large or inoperable primary (T3–4) or (ii) palpable LN > 3 cm or (iii) fixed LNs or suspicion of extranodal extension on imaging or (iv) pelvic nodal involvement Primary tumour 24/33 (73%), recurrent tumour 9/33 (27%) T stage: cT1 3/33 (9%), cT2 9/33 (27%), cT3 13/33 (39%), cT4 4/33 (12%), cTX 4/33 (12%) N stage: cN0 3/33 (9%), cN1 0/33, cN2 3/33 (9%), cN3 27/33 (88%) Clinical stage: IIb 1/33 (3%), IIIb 3/33 (9%), IV (T4, Nany, M0 and/pr Tany, N3, M0) 29/33 (88%)	If feasible, primary tumour was resected before CRT initiation Radiation dose 59.4 Gy in 33 daily fractions of 1.8 Gy to primary tumour and involved LNs over 6.5 wks; electively treated LN received 49.5 Gy in 33 fractions of 1.5 Gy over same treatment time; simultaneous integrated boost VMAT CRT field: primary tumour 1/33 (33%), LNs 21/33 (64%), both LNs + primary 11/33 (33%) Concurrent chemotherapy: mitomycin on day 1 + capecitabine on radiation days Residual/recurrent disease underwent salvage surgery if feasible FDG-PET/CT response acquired 6 wks post-CRT	41	Primary: PFS Secondary: 2-year PFS, 1- and 2-year OS, local control rates, ORR (FDG-PET/CT-based response), CTCAE toxicity/adverse events	Overall, 1-yr and 2-yr PFS (95% CI) 34% (22–55%) and 31% (19–52%); 1-yr and 2-yr OS (95% CI) 73% (60–90%) and 46% (32–67%) ORR = 24/33 (73%); CR = 13/33 (39%); PR = 11/33 (33%); SD = 1/33 (3%); PD = 5/33 (15%); not evaluable = 3/33 (9%) In-field control rate = 52% Overall, 9/33 underwent salvage surgery + 8/33 later surgery (total 52%) Overall, 11/33 (33%) had ≥ 1 Grade 3 treatment-related adverse events; no Grade 4/5 adverse events. Most frequent adverse event was moderate radiation dermatitis in 28/33 (85%); one discontinued after 11 fractions (skin toxicity)
Necchi et al. (2017) [40]	Retrospective cohort study	Group 1: NAC, n = 94 Group 2: AC, n = 78 Group 3: NAC + AC, n = 21 Unknown chemo. timing, n = 8	Median age 62 yrs (range 35–87) SCC, n = 144/201; sarcomatoid, n = 8/201; verrucous, n = 19/201; warty, n = 5/201; basaloid, n = 1/201; papillary, n = 16/201 ECOG PS: 0–1 (82.1%), ≥2 (4.5%), unknown (13.4%) Staging: Tany, cN+ (n = 162 [80.6%]; NAC = 77/94; AC = 60/78; NAC + AC = 17/21); T3-4, cN0 (n = 39 [19.4%]; NAC = 17/94; AC = 18/78; NAC + AC = 4/21). No metastatic disease in any patient at baseline	Concomitant radiotherapy, n = 43/201: NAC (12/94 [12.8%]), AC (28/78 [35.9%]), NAC + AC (3/21 [14.3%]) (*p* < 0.001 for difference) See Table 1. for details on chemotherapy	Not reported	OS RFS	Prior radiation of primary tumour was not significantly associated with RFS (HR 0.69, 95% CI 0.28–1.67, *p* = 0.41) or OS (HR 1.02, 95% CI 0.42–2.47, *p* = 0.97) on univariate analysis Concomitant radiotherapy (with NAC or AC) not associated with RFS (HR 0.80, 95% CI 0.41–1.59, *p* = 0.53) or OS (HR 0.74, 95% CI 0.36–1.51, *p* = 0.40) on univariable analysis
Ravi et al. (1994) [21]	Retrospective cohort study	Neoadjuvant radiotherapy, n = 45	Penile cancer histological subtype frequency not reported Indication for neoadjuvant radiotherapy prior to LND: inguinal nodes ≥ 4 cm in size not fixed (n = 33), nodes of any size fixed to skin but mobile (n = 12) Baseline characteristics not detailed for each treatment setting	External beam radiotherapy: total dose 50–60 Gu/5–6 weeks For inguinal LN involvement, preoperative radiotherapy dose 40 Gy/4 weeks	83	ORR NB: PR = ≥50% decrease in size of measurable disease	ORR after neoadjuvant radiotherapy = 17.3%; CR = 2%; PR = 15.4%; <PR = 82.7% On LND specimens post-neoadjuvant CRT for mobile inguinal LN ≥ 4 cm, only 8% had perinodal infiltration and only 3% had postoperative groin recurrences DFS and OS not reported for neoadjuvant subgroup

Abbreviations: AC = adjuvant chemotherapy. CR = complete response. CRT = chemoradiotherapy. CTCAE = Common Terminology Criteria for Adverse Events. DFS = disease-free survival. ECOG = Eastern Cooperative Oncology Group. FDG-PET/CT = 18F-fluordeoxyglucose positron emission tomography/computed tomography. 5-FU = 5-fluorouracil. GFR = glomerular filtration rate. IMRT = intensity-modulated radiation therapy. NAC = neoadjuvant chemotherapy. LN = lymph node. (P)LND = (pelvic) lymph node dissection. OS = overall survival. PFS = progression-free survival. PR = partial response. PS = performance status. SCC = squamous cell carcinoma. TIP = paclitaxel, ifosfamide, cisplatin. VMAT = volumetric arc therapy. WHO = World Health Organisation.

**Table 3:** Summary of studies on immunotherapy with or without other systemic therapy for penile cancer with potential neoadjuvant application.

Study	Design	Study Population Size	Disease Characteristics	Treatment Details	Median Follow-Up, Months	Outcome Measures	Results
An et al. (2025) [61] Clinicaltrials.gov NCT04475016	Phase II, single-arm prospective trial	Neoadjuvant combination systemic therapy with immunotherapy, n = 29 No comparator	Median age: 57 yrs (range 31–71 yrs) ECOG PS: 0 (1/29 [3.4%]), 1 (19/29 [65.6%]), 2 (9/29 [31%]) High-risk, locally advanced penile SCC in all (cTx-4N3M0) Clinical T stage: Tx (12/29 [41.4%]), T1–3 (4/29 [13.8%]), T4 (13/29 [44.8%]) Clinical N stage: N3 29/29 (100%), inguinal LN mets 10/29 (34.5%), pelvic LN mets 19/29 (65.5%) Previous local therapy: absent 6/29 (20.7%), partial penectomy 19/29 (65.5%), total penectomy 2/29 (6.9%), unilateral ILND 7/29 (21.4%), bilateral ILND 6/29 (20.7%) Histopathologic stage: I (8/29 [27.6%]), II (16/29 [55.52%]), III (5/29 [17.2%])	Triple combination regimen toripalimab (anti-PD-1 mAb) day 1, nimotuzumab (anti-EGFR mAb) day 1, taxol-based chemotherapy IV (albumin-bound paclitaxel day 1, cisplatin day 1–3, ifosfamide day 1–3), administered every 3 weeks to max. four cycles or until dose-limiting toxicity or disease progression or patient withdrawal G-CSF allowed after 24–48 h of chemotherapy Consolidative surgery done for medically fit patients with resectable residual lesions (aiming for R0); types of surgery included (partial or total) penectomy, uni- or bilateral ILND ± PLND; surgery extent based on pre-neoadj. radiological burden of disease and need for consolidative resection to remove all sites of suspected residual disease	40	Primary: pCR rate (ypTis/T0N0M0) Secondary: ORR (RECIST criteria) OS PFS NCI-CTCAE toxicity Postop complications (Clavien–Dindo) Radiologic assessment performed every 6 wks with CT or MRI according to RECIST v1.1	Positive trial as primary endpoint met: pCR rate 48.3% (14/29, 95% CI 29.4–67.5%), powered to detect pCR 30% with null hypothesis 10% pCR based on TIP trial [30] All operated patients (n = 24/29 [82.8%]) achieved R0 resection ORR after neoadjuvant combination = 82.8% (24/29, 95% CI 64.2–94.2%) after four cycles of combination regimen; CR = 10.3% (3/29, 95% CI 2.2–27.4%); PR = 72.4% (21/29, 95% CI 52.8–86.6%); SD = 10.3% (3/29); PD = 6.9% (2/29) Median PFS and OS NR; at 40 mo f/u, 6/24 (25%) operated patients had radiologic recurrence, 9/29 (31%) patients died of disease progression; 2-yr PFS 65.5% (95% CI 48.3–82.7%), 2-yr OS 72.4% (95% CI 56.1–88.7%) pCR associated with significantly longer PFS (median NR vs. 13.27 mos, HR 0.105, 95% CI 0.020–0.567, *p* = 0.01) and OS (median NR vs. NR, HR 0.148, 95% CI 0.024–0.909, *p* = 0.048) Radiologic clinical responders (CR + PR) had significantly longer PFS (median NR vs. 2.47 mos, HR 0.067, 95% CI 0.005–0.862, *p* < 0.001) and OS (median NR vs. 10.5 mos, HR 0.063, 95% CI 0.005–0.776, *p* < 0.001) than non-responders (SD + PD) Any grade treatment-related adverse events occurred in all patients; Grade 3/4 in 12/29 (41.4%), most commonly neutropenia (7/29), anaemia (3/29), febrile neutropenia (2/29), rash (2/29); most common potentially immune-related adverse events Grade 1/2 hypothyroidism (5/29), hyperthyroidism (3/29), adrenal insufficiency (2/29); dose reduction due to Grade 3/4 toxicity in 3/29; 1/29 discontinued due to Grade 3 asthenia
PRIAM trial [57] Clinicaltrials.gov: NCT06353906	Phase II, single-arm prospective trial	Currently recruiting Planned neoadjuvant enrolment, n = 27 No comparator	Locoregionally advanced penile SCC Clinical stage cTxN1-3 (node-positive disease) and considered suitable for resection (post-induction) Patients with recurrence in lymph nodes included if have not received prior systemic treatment; patients with cN1 are only included in case of central nodal necrosis and/or an irregular nodal border, or node > 3 cm on CT ≤2 supraregional/distant metastases allowed if local treatment (i.e., irradiation or resection) is feasible	Induction systemic therapy with neoadjuvant intent: carboplatin + paclitaxel chemotherapy combined with pembrolizumab (anti-PD-1 mAb) Three cycles of carboplatin/paclitaxel; pembrolizumab on cycles 1 and 3 (21-day cycles), followed by reassessment imaging Local consolidation: surgical resection of residual disease if feasible post-induction (partial/total penectomy + ILND +/− PLND) within 3–9 weeks of last induction cycle Postoperative pembrolizumab continued for seven cycles every 6 weeks	Not yet reported	Primary: pCR rate Secondary: Grade 3–4 toxicities (CTCAE), PFS and OS at 2 yrs post-surgery, correlation between clinical endpoints and high-risk HPV and PD-L1 immunohistochemistry, QoL Response to induction assessed by CT scan 2 wks after last cycle	Trial ongoing (not yet reported)
Clinicaltrials.gov: NCT06415318 [58]	Phase II, single-arm prospective trial	Currently recruiting Planned neoadjuvant enrolment, n = 25 No comparator	Locally advanced penile SCC: T4, any N stage OR any T stage, N3 ECOG 0–2 Measurable disease required (≥1 measurable lesion per RECIST 1.1) No prior chemotherapy (unless relapse > 12 mth after last chemotherapy)	Neoadjuvant combination chemoimmunotherapy given q21 days until surgery, progression, or unacceptable toxicity: toripalimab (anti-PD-1 mAb) day 1, paclitaxel day 1, cisplatin days 1–3, ifosfamide days 1–3 Number of cycles not specified in protocol	Not yet reported	Primary: ORR (RECIST criteria) at 6 weeks Secondary: pCR rate, EFS, OS, NCI-CTCAE toxicity	Trial ongoing (not yet reported)
Chen et al. (2022) [60] Trial registration: ChiECRCT20210503	Phase II, single-arm prospective trial	Currently recruiting Planned neoadjuvant enrolment, n = 34 No comparator	Locally advanced penile SCC: Tx, N2-N3, M0 Inguinal and/or pelvic LN mets confirmed by percutaneous biopsy Primary tumour resection prior to LN biopsy ECOG PS 0–1	Neoadjuvant chemoimmunotherapy administered q21-days for four cycles prior to LND: camrelizumab (anti-PD-1 mAb), paclitaxel, cisplatin, ifosfamide Neoadjuvant treatment intent: tumour down-staging to enable radical LND	Not yet reported	Primary: pCR rate Secondary: EFS, OS, ORR (RECIST criteria), DCR, CTCAE toxicity	Trial ongoing (not yet reported) Target sample size powered to detect pCR 34% (power 80%, one-sided alpha 0.05)
Hahn et al. (2021) [59] ClinicalTrials.gov: NCT02721732	Case series from phase II, single-arm basket trial	Penile SCC cohort, n = 3 No comparator	Age: 66, 71, 76 yrs SCC in all Recurrent, locally advanced or metastatic Prior progression on platinum-based chemotherapy MSI-H (loss of MLH1/PMS2) in 1/3, MSS in 2/3	Salvage single-agent pembrolizumab (anti-PD-1 mAb), 200 mg IV every 3 weeks Surgical consolidation if response	Not reported (range 3.8–38.7 mo)	ORR (RECIST criteria) Duration of response OS CTCAE toxicity Correlation with MSI	ORR after immunotherapy = 1/3 (33%); PR = 1/3 with MSI-H tumour; PD = 2/3 with MSS tumours Durability: responding MSI-H patient achieved PR after six cycles, underwent surgical consolidation, disease-free at 38.7 months No Grade ≥ 3 immune-related adverse events; Grade 2 hypothyroidism, rash, and anorexia reported

Abbreviations: CI = confidence interval. (p)CR = (pathologic) complete response. DCR = disease control rate. ECOG = Eastern Cooperative Oncology Group. EFS = event-free survival. G-CSF = granulocyte colony-stimulating factor. HR = hazard ratio. LN = lymph node. (I)/(P)LND = (inguinal)/(pelvic) lymph node dissection. mAb = monoclonal antibody. MSI = microsatellite instability. MSS = microsatellite stability. NCI-CTCAE = National Cancer Institute Common Terminology Criteria for Adverse Events. NR = not reached. ORR = objective response rate. OS = overall survival. PD = progressive disease. PFS = progression-free survival. PR = partial response. PS = performance status. QoL = quality of life. RECIST = Response Evaluation Criteria in Solid Tumors version 1.1. SCC = squamous cell carcinoma. SD = stable disease. TIP = paclitaxel, ifosfamide, cisplatin.

**Table 4:** Summary of studies assessing molecularly targeted therapy with potential application to neoadjuvant treatment of penile cancer.

Study	Design	Study Population Size	Disease Characteristics	Treatment Details	Median Follow-Up, Months	Outcome Measures	Results
Necchi et al. (2018) [64] ClinicalTrials.gov NCT01728233	Phase II, single-arm, prospective trial	First-line TKI, n = 28 No comparator	Median age: 63.5 yrs (IQR 53.5–67.5) Pure penile: SCC 96.4%; sarcomatoid variant 3.6% Locally advanced nodal disease (N2-3): 20/28 (71%); visceral metastatic disease (M1): 8/28 (29%) Nodal burden: bilateral inguinal LN 60.7%; pelvic LN 50% No prior systemic therapy permitted Prior PLND 6/28 (21.4%); prior ILND 17/28 (60.7%) ECOG PS: 0–1	Dacomitinib (pan-HER TKI) 45 mg PO daily Treatment continued until radiologic progression or unacceptable toxicity Response assessment: CT + FDG/PET every 8 weeks Surgical consolidation offered to responding patients A total of 18/28 underwent LND after dacomitinib exposure	19.8	Primary: ORR (RECIST criteria) Secondary: PFS, OS, CTCAE toxicity Exploratory: EGFR/ERBB2 amplification, HPV status, NGS-defined genomic correlates	ORR = 32.1% (80% credibility interval 21–43%); CR = 1/28 (3.6%); PR = 8/28 (28.6%); SD = 46.4%; PD = 21.4% Median OS: 13.7 mos (95% CI 9.9—NR) Median PFS: 4.1 mos (95% CI 3.1—NR) Locally advanced (non-metastatic) subgroup: median OS 20 mos (95% CI 11.1—NR) No pCR Toxicity: Grade 3 adverse events 10.7% (skin toxicity only); no treatment-related deaths TERT mutations in responders only (60%) PI3K/mTOR mutations in 42.9% responders vs. 8.3% non-responders EGFR amplification found in four patients (equally responders and non-responders)
Necchi et al. (2017) [63] Clinical trial identification: EudraCT number 2017-001963-19	Phase II, single-arm, prospective trial	Currently recruiting Planned total enrolment, n = 37 No comparator	Penile SCC Clinical stage: locally advanced nodal disease (cN2-3) or metastatic disease (M1) Prior systemic therapy not permitted	Cabozantinib 60 mg PO daily Administered until surgery Response assessment FDG-PET/CT, every 2 months Responding locally advanced cN2-3 patients eligible for consolidative LND	Not yet reported	Primary: ORR (RECIST criteria) Secondary: safety, PFS, OS, pathological response	Trial not yet reported
Carthon et al. (2014) [62]	Retrospective cohort study	EGFR-targeted therapy, n = 24 No comparator	Median age: 59 yrs (IQR 50.3–68.3) SCC in all (23/24 penile; 1/24 scrotal) cT4 or any T with cN2/3 or M1 Metastatic burden at EGFR therapy start: 12/24 (50%) had distant visceral/bony mets; 12/24 locally advanced nodal masses Heavily pre-treated: 22/24 (91.7%) ≥ 1 prior systemic chemotherapy line; 8/24 ≥ 2 prior lines EGFR immunohistochemistry: 13 tested, all positive	EGFR-targeted agent alone (cetuximab/erlotinib/gefitinib), n = 8 Cetuximab + platinum (cisplatin n = 12; carboplatin n = 1), n = 13 Cetuximab + TIP, n = 3 Three patients underwent consolidative surgery after response to EGFR-targeted therapy in neoadjuvant setting (cetuximab + cisplatin n = 2; cetuximab + TIP n = 1)	6.8	OS and PFS from start of EGFR-targeted therapy ORR (RECIST criteria) CTCAE toxicity	Median PFS: 2.6 mo (range 0.4–9.2) Median OS: 6.8 mo (range 0.5–47.4) Individual OS in neoadjuvant/consolidative surgery group: 15.0, 37.2, and 47.4 mo Significantly longer OS after neoadjuvant + consolidative surgery vs. no consolidative surgery median OS 5.9 mo (log-rank test *p* = 0.028) Overall, 2/3 who had neoadjuvant EGFR-targeted therapy + consolidative surgery had long-term disease-free survival (44 mos and 35 mos) ORR = 25% (6/24): PR 1/5 (after cetuximab alone) and 3/12 (cetuximab + cisplatin) and 2/3 (cetuximab + TIP); no objective responses to gefitinib or erlotinib (small molecule inhibitors) Toxicity: well-tolerated; Grade 1–2 rash most common (70.8%); Grade 3–4 events after cetuximab uncommon (cellulitis 1/21, thrombocytopenia 1/21, bronchospasm 1/21)

Abbreviations: CI = confidence interval. CR = complete response. CTCAE = Common Terminology Criteria for Adverse Events. CSS = cancer-specific survival. DSS = disease-specific survival. DFS = disease-free survival. ECOG PS = Eastern Cooperative Oncology Group Performance Status. EGFR = epidermal growth factor receptor. ENE = extranodal extension. 5-FU = 5-flurouracil. HR = hazard ratio. ICI = immune checkpoint inhibitor. IQR = interquartile range. LN = lymph node. (I)/(P)LND = (inguinal)/(pelvic) lymph node dissection. Mos = months. NAC = neoadjuvant chemotherapy. NED = no evidence of disease. ORR = objective response rate. OM = overall mortality. OS = overall survival. PD = progressive disease. PFS = progression-free survival. PR = partial response. QoL = quality of life. SCC = squamous cell carcinoma. SD = stable disease. TIP = taxane, ifosfamide, cisplatin. TKI = tyrosine kinase inhibitor.

**Table 5:** Summary of guidelines and strength of recommendations for neoadjuvant treatment of penile cancer.

Guideline	Recommendation on Neoadjuvant Treatment Approach	Preferred/Named Neoadjuvant Regimen(s)	Strength/Level of Evidence
EA U-ASCO updated guideline (2026) [65]	Offer neoadjuvant chemotherapy (NAC) using a cisplatin- and taxane-based combination to chemotherapy-fit patients with pelvic lymph node involvement or those with extensive inguinal involvement (cN3) in preference to upfront surgery. Offer chemotherapy as an alternative approach to upfront surgery to selected patients with bulky mobile inguinal nodes or bilateral disease (cN2) who are candidates for cisplatin and taxane-based chemotherapy. Strong recommendation to offer completion surgery to patients responding to NAC in whom resection is feasible due to long-term survival associated with consolidative surgery in responders.	Limited available evidence favours a cisplatin- and taxane-based combination (doublet or triplet) as the preferred preoperative approach (TIP, TPF, or PF).	Weak recommendation—supporting evidence is low and based mainly on phase II and retrospective data
ESMO-EURACAN (2024) [6]	All chemotherapy-eligible patients with locally advanced (T4), inoperable primary penile cancer or those with ulcerated or fixed inguinal, or pelvic lymph node disease (cN3) should be offered neoadjuvant chemotherapy. Adjuvant instead of neoadjuvant chemotherapy recommended for cN1-2 (palpable LN) disease that has positive fine-needle biopsy and high-risk features on subsequent radical inguinal lymph node dissection (≥2 positive LNs, ENE, metastasis diameter > 30 mm)—data less clear in this setting.	Up to four cycles of a triplet regimen such as TIP or TPF.	Level III = prospective cohort studies Grade B = strong or moderate evidence for efficacy but with a limited clinical benefit, generally recommended
NCCN v1.2026 [9]	Palpable inguinal lymph nodes → imaging of chest/abdomen/pelvis. → Unilateral inguinal LN (s) < 4 cm (mobile) [non-bulky inguinal LNs] with either high-risk primary (T1, high-grade, LVI, PNI, >50% poorly differentiated) or low-risk primary but positive percutaneous LN biopsy, should be considered for NAC followed by ILND. → Unilateral inguinal LN (s) ≥ 4cm (mobile) with positive percutaneous LN biopsy should be considered for NAC followed by ILND (preferred), with consideration of PLND; RT or chemo/RT are less preferred alternatives. → Unilateral LN (s) (fixed) or bilateral LNs (fixed or mobile) with positive percutaneous LN biopsy or excisional biopsy should be offered NAC and those not eligible for NAC should be offered ILND and PLND or RT or chemo/RT. → Pelvic lymphadenopathy with positive percutaneous LN biopsy and surgical candidate should be offered NAC; if negative biopsy, manage as per inguinal LN status.	TIP Platinum-based (TIP) NAC favoured over taxane-based (TPF) due to higher response rates and lower rates of grade three or higher AEs for platinum-based chemotherapy in a stratified sub-analysis.	Category 2A—based upon lower-level evidence than randomised phase 3 trials but there is uniform NCCN consensus with ≥85% support of the Panel that the intervention is appropriate TIP is “Preferred” = intervention based on superior efficacy, safety, and evidence compared to alternatives
French AFU Cancer Committee (2024–2026) [11]	T4 disease—NAC should be considered along with total amputation and perineal urethrostomy if tumour is removable. Massive pelvic or fixed inguinal LN disease (cN2-3) should be considered for NAC, with consolidative inguinal lymphadenectomy only for those who respond to chemotherapy (due to poor prognosis with surgery in non-responders).	Preferred regimen not specified, but guideline states that chemotherapy must include a combination of three compounds comprising at least one platinum salt and a taxane, together with either 5-fluorouracil (TPF protocol), which is considered poorly tolerated, or ifosfamide (TIP protocol).	“Low-grade” recommendation for cN3 disease “High-grade” recommendation for T4 disease (Grades not defined in text)

Abbreviations: TIP = paclitaxel + ifosfamide + cisplatin. TPF = docetaxel, cisplatin, 5-fluorouracil. PF = cisplatin/5-fluorouracil. LN = lymph node. ENE = extranodal extension. (I)LND = (inguinal) lymph node dissection. RT = radiotherapy. PNI = perineural invasion. LVI = lymphovascular invasion.

**Table 6:** Clinical trials in progress investigating neoadjuvant treatment of penile cancer.

Trial	Design/Phase	Treatment Modality	Population	Interventions	Endpoint (s)	Status/Estimated Study Completion Date/Relevance
**InPACT NCT02305654** [8]	International phase III, Bayesian, multi-stage randomised trial	Chemotherapy (TIP) +/− radiotherapy (IMRT)	Clinically node-positive penile SCC	ILND alone vs. TIP NAC + ILND vs. neoadjuvant chemoradiotherapy + ILND, with subsequent pelvic management randomisation in high-risk patients	**Primary**: OS **Secondary**: DSS, DFS, freedom from locoregional recurrence and distant metastasis, feasibility, toxicity, surgical complications, QoL	Recruiting Estimated completion: 30 November 2027 Pivotal practice-defining perioperative treatment trial, evaluating optimal perioperative treatment sequencing and comparative activity of surgery, NAC, and CRT
**PRIAM NCT06353906** [57]	Phase II, single-arm	Immunotherapy, anti-PD-1 mAb	cTxN1–3 locoregionally advanced resectable penile SCC	Induction carboplatin/paclitaxel + pembrolizumab followed by response-adapted surgery and adjuvant pembrolizumab	**Primary**: pCR **Secondary**: CTCAE, PFS, correlation with HPV and PD-L1 biomarkers, QoL	Recruiting Estimated completion: 14 January 2028 Directly investigating neoadjuvant application of chemoimmunotherapy
**NCT06415318** [58]	Phase II, single-arm	Immunotherapy, anti-PD-1 mAb	Locally advanced penile cancer (T4 any N or any T N3)	TIP plus toripalimab before surgery	**Primary**: ORR **Secondary**: pCR, EFS, OS, CTCAE toxicity	Recruiting Estimated completion: 1 December 2027 Directly investigating neoadjuvant application of chemoimmunotherapy
**ChiECRCT20210503** [60]	Phase II, single-arm prospective trial	Immunotherapy, anti-PD-1 mAb	Locally advanced penile SCC: Tx, N2-N3, M0 Inguinal and/or pelvic LN mets confirmed by percutaneous biopsy ECOG PS 0–1	Neoadjuvant chemoimmunotherapy administered q21-days for four cycles prior to LND: camrelizumab (anti-PD-1 mAb), paclitaxel, cisplatin, ifosfamide	**Primary:** pCR **Secondary**: EFS, OS, ORR (RECIST criteria), DCR, CTCAE toxicity	Recruiting Estimated completion: not reported Directly investigating neoadjuvant application of chemoimmunotherapy
**EudraCT 2017-001963-19** [63]	Phase II, single-arm, prospective trial	Molecularly targeted therapy, multi-tyrosine kinase inhibitor (anti-MET, anti-VEGFR, anti-AXL)	Penile SCC Clinical stage: locally advanced nodal disease (cN2-3) or metastatic disease (M1)	Cabozantinib 60 mg PO daily Administered until surgery Response assessment FDG-PET/CT, every 2 months Responding locally advanced cN2-3 patients eligible for consolidative LND	**Primary**: ORR **Secondary**: PFS, OS, pathological response, safety	Recruiting Estimated completion: not reported Directly investigating neoadjuvant application of molecularly targeted therapy
**SMART NCT06161532** [105]	Phase II, non-randomised two-arm study	Molecularly targeted therapy, TROP-2-directed antibody and topoisomerase inhibitor conjugate Immunotherapy, anti-PD-L1	Rare GU tumours including penile SCC, largely advanced/unresectable or metastatic	Sacituzumab govitecan (antibody–drug conjugate) ± atezolizumab (anti-PD-L1)	**Primary**: ORR **Secondary**: duration of response, OS, PFS, CBR, safety	Recruiting Estimated completion: 1 November 2028 Basket study involving multiple GU tumour types; not pure neoadjuvant trial but relevant translational platform for future perioperative targeted/immunotherapy combinations given inclusion of preoperative application to locally advanced, non-metastatic setting

**Abbreviations:** SCC = squamous cell carcinoma. (I)LND = (inguinal) lymph node dissection. OS = overall survival. DSS = disease-specific survival. DFS = disease-free survival. QoL = quality of life. NAC = neoadjuvant chemotherapy. CRT = chemoradiotherapy. pCR = pathological complete response. EFS = event-free survival. ORR = objective response rate. GU = genitourinary. CTCAE = Common Terminology Criteria for Adverse Events. HPV = human papilloma virus. mAb = monoclonal antibody. DCR = disease control rate. TIP = paclitaxel + ifosfamide + cisplatin. IMRT = intensity-modulated radiation therapy. CBR = clinical benefit rate (% of patients achieving complete response, partial response, or stable disease while on treatment).

## Data Availability

Not applicable; this is a systematic review with no original data.

## Appendix A

**Table A1:** Population, Intervention, Comparator, Outcome and Method (PICOM) eligibility criteria for included studies.

P (Population)	Include: • Adults ≥ 18 years. • Males. • Histologically confirmed diagnosis of penile cancer. • Curative treatment intent. • Planned for neoadjuvant treatment prior to curative resection of penile cancer. • Any histological subtype of penile cancer.	Exclude: • Children aged < 18 years. • Advanced penile cancer including metastatic disease and recurrent disease post-definitive treatment. • Penile cancer stage or treatment intent not specified.
I (Intervention)	Include: • Any neoadjuvant chemotherapy regimen. • Any neoadjuvant immunotherapeutic regimen. • Any neoadjuvant molecularly targeted regimen. • Any neoadjuvant radiotherapy regimen. • Any other treatment directed at penile cancer in the neoadjuvant setting.	Exclude: • Alternative medicine/naturopathic strategies. • Only assessing adjuvant treatment post-definitive treatment.
C (Comparator)	Include: • Placebo. • Other systemic therapies or radiotherapy directed at penile cancer in the neoadjuvant setting. • Patients undergoing adjuvant treatment strategies (post-definitive treatment). Single-arm studies without comparator groups were also included for evaluation of oncological efficacy.	Exclude: • Studies comparing outcomes between groups of different cancer types, e.g., squamous cell carcinoma (SCC) of penile cancer versus SCC of other location.
O (Outcome)	Include—primary oncological outcomes: • Objective response rate (ORR). • Pathological complete response rate (PCR). • Overall survival (OS). • Progression free survival (PFS). Include—secondary outcomes assessing safety: • Incidence of adverse event. • Incidence of dose-limiting toxicity.	Exclude: • Nil specific exclusions based on outcomes.
M (Method)	Include: • Randomised controlled trials. • Non-randomised clinical trials. • Observational cohort studies (prospective or retrospective). • Case–control studies. • Case series. Clinical trial protocols were included even when the trial was still accruing and not yet complete.	Exclude: • Case reports. • Cross-sectional studies. • Systematic or narrative reviews. Clinical trial protocols were excluded when the trial was stopped prior to completion for any reason. Reasons for withdrawal of clinical trial protocols were recorded.
